# Deposition of Antioxidant and Cytocompatible Caffeic Acid-Based Thin Films onto Ti6Al4V Alloys through Hexamethylenediamine-Mediated Crosslinking

**DOI:** 10.1021/acsami.3c05564

**Published:** 2023-06-08

**Authors:** Maria L. Alfieri, Giacomo Riccucci, Sara Ferraris, Andrea Cochis, Alessandro C. Scalia, Lia Rimondini, Lucia Panzella, Silvia Spriano, Alessandra Napolitano

**Affiliations:** † Department of Chemical Sciences, University of Naples Federico II, Via Cintia 21, Naples I-80126, Italy; ‡ Politecnico di Torino, Corso Duca degli Abruzzi 24, Torino 10129, Italy; § Department of Health Sciences, Center for Translational Research on Autoimmune and Allergic Diseases CAAD, University of Piemonte Orientale, Corso Trieste 15/A, Novara, Novara 28100, Italy; ∥ Interdipartimental Laboratory PolitoBIOMedLab, Politecnico di Torino, Torino 10129, Italy

**Keywords:** caffeic acid, hexamethylenediamine, titanium alloys, thin coatings, antioxidant properties, cytocompatibility

## Abstract

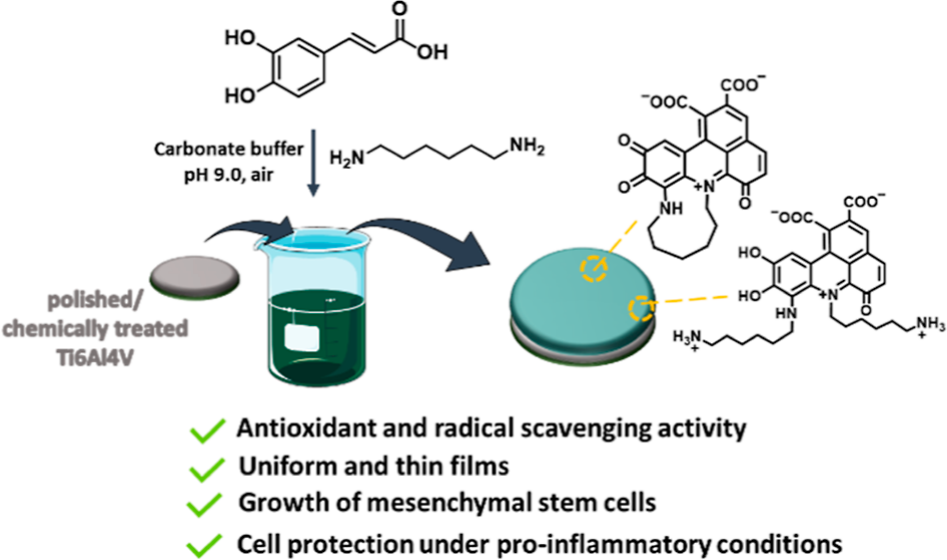

A promising approach for advanced bone implants is the deposition on titanium surfaces of organic thin films with improved therapeutic performances. Herein, we reported the efficient dip-coating deposition of caffeic acid (CA)-based films on both polished and chemically pre-treated Ti6Al4V alloys by exploiting hexamethylenediamine (HMDA) crosslinking ability. The formation of benzacridine systems, resulting from the interaction of CA with the amino groups of HMDA, as reported in previous studies, was suggested by the yellow/green color of the coatings. The coated surfaces were characterized by means of the Folin–Ciocalteu method, fluorescence microscopy, water contact angle measurements, X-ray photoelectron spectroscopy (XPS), zeta-potential measurements, and Fourier transform infrared spectroscopy, confirming the presence of a uniform coating on the titanium surfaces. The optimal mechanical adhesion of the coating, especially on the chemically pre-treated substrate, was also demonstrated by the tape adhesion test. Interestingly, both films exhibited marked antioxidant properties (2,2-diphenyl-1-picrylhydrazyl and ferric reducing antioxidant power assays) that persisted over time and were not lost even after prolonged storage of the material. The feature of the coatings in terms of the exposed groups (XPS and zeta potential titration evidence) was apparently dependent on the surface pre-treatment of the titanium substrate. Cytocompatibility, scavenger antioxidant activity, and antibacterial properties of the developed coatings were evaluated. The most promising results were obtained in the case of the chemically pre-treated CA/HMDA-based coated surface that showed good cytocompatibility and high reactive oxygen species’ scavenging ability, preventing their intracellular accumulation under pro-inflammatory conditions; moreover, an anti-fouling effect preventing the formation of 3D biofilm-like bacterial aggregates was observed by scanning electron microscopy. These results open new perspectives for the development of innovative titanium surfaces with thin coatings from naturally occurring phenols for bone contact implants.

## 1 Introduction

Titanium and its alloys are among the most widely employed materials for bone implants due to their outstanding mechanical properties, biocompatibility, cost-effectiveness, and excellent durability.^1,2^ Among titanium alloys, Ti6Al4V, which can be fabricated by additive manufacturing technologies, has the highest relevance and largest use for orthopedic implants. The ordinary need for novel implants for bone and joint replacement or to support damaged bones has recently motivated research on the optimization of the surface properties of Ti6Al4V in order to promote tissue healing even in adverse conditions such as inflammation or infection.^3^ Improvement of osteointegration and antibacterial or anti-microfouling bioactivity of the materials has recently been gained, for example, by bioactive coatings,^4−6^ chemical/electrochemical treatments to get bioactive oxide layers,^7,8^ and modification of surface topography to get porous structures favoring the proliferation of human bone marrow stromal cells as well as the in-growth of the mature osteoblasts and the colonization of the implant by the progenitor cells migrating from the surrounding healthy tissue.^9−11^ Moreover, the ability of the implantable materials to prevent bacterial colonization and biofilm formation is of crucial importance since infections still represent a major issue in orthopedics; for example, in their clinical revisions, Hardes et al.^12^ and Wafa et al.^13^ reported an infection incidence of 17.6 and 22.4%, respectively, for patients carrying Ti implants devoid of antibacterial agents.

In this regard, the use of natural polyphenols to further improve the interfacial features of Ti6Al4V surfaces may represent a promising approach in view of the extremely versatile chemistry and unparalleled properties of these compounds including antioxidant, anti-inflammatory, anti-cancer, and anti-microbial activity as well as the positive effects on bone healing.^14,15^ Their bio-adhesiveness, large availability, and cost-effectiveness represent additional advantages.^16,17^ So far, commercially available polyphenols including epigallocatechin gallate, gallic acid,^18,19^ or polyphenols containing natural extracts (e.g., green tea extracts, grape pomace)^20,21^ have been directly grafted onto implantable materials, with promising effects on osteoblast growth and bone mineralization while enhancing osseointegration activity. It was also previously demonstrated that when polyphenols (e.g., gallic acid, caffeic acid (CA), red grape skin extract) are grafted onto bioactive glasses as well as on stainless steel substrates or magnesium alloys, wettability and cytocompatibility are improved, corrosion is well mitigated, and they could be effective as bone substitutes in cancer treatment.^22,23^ In combination with biocompatible and biodegradable polymers, catechin and resveratrol showed, for example, a great potential for wound dressing due to the antibacterial activity, breathability, and ability to absorb excess exudate of biofilms.^24^ Moreover, several studies reported the improvement of hydrophilicity, cytocompatibility, antibacterial and antioxidant properties, as well as the enhancement of osteogenesis *in vitro* and bone formation *in vivo* of titanium implants by coating with polydopamine and/or tannic acid.^25^ However, poor attention has been so far devoted to the design and synthesis of bioinspired polyphenol-based systems that might offer advantages with respect to natural polyphenols in terms of stability, antioxidant ability, adhesion properties, presence of several functional groups allowing for their anchoring to different substrates, and controlled release in the target site, which makes them ideal candidates for surface coating on titanium implants. Thin organic natural coatings applied on pre-treated titanium substrates will in fact allow for the combination of topographical and chemical/biological stimuli on the same surface because the nano-/micro-texture of the titanium substrate will not be masked by the thin coatings.

**Scheme 1:**
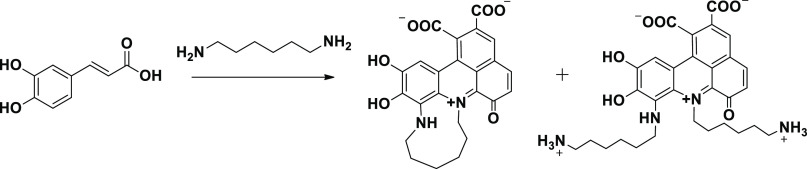
Overview of the Main Structural Components of CA/HMDA Films, Depicted in the Zwitterionic Form, Based on ^13^C NMR and ^15^N NMR and LDI-MS Analysis^29^

Interesting opportunities for the design and development of functional biomaterials and systems come from the oxidatively induced crosslinking reaction of catechols with amines.^26−28^ In particular, the combination of CA, a cheap, non-toxic, and easily accessible natural catechol, with a long aliphatic chain diamine, hexamethylenediamine (HMDA), provides the basis for a novel, cost-effective, and simple methodology to achieve a biocompatible organic thin film, which can adhere to a broad range of surfaces.^29^ As demonstrated by ^13^C and ^15^N cross-polarization magic angle spinning nuclear magnetic resonance (CP-MAS NMR) combined with attenuated total reflectance (ATR)/Fourier transform-infrared spectroscopy (FT-IR) and laser desorption/ionization (LDI)/matrix-assisted laser desorption/ionization mass spectrometry (MALDI-MS) analysis, the CA/HMDA coating consisted of a mixture of trioxygenated carboxylated benzacridinium derivatives resulting from the covalent incorporation of the amine during CA oxidative polymerization.^30,31^ Interestingly, the coating exhibited a pH-dependent chromophore, metal-chelating properties, dye-adsorbing ability, and, most importantly, provided an excellent cytocompatible platform for growing embryonic stem cells (Scheme 1).^29^

Although several works report the grafting or coating of different catechols on titanium surfaces, CA has been poorly investigated.^18−22^ Based on the outstanding properties observed for the CA/HMDA films and inspired by their promising biological response, in this work, the CA/HMDA film-forming ability already observed on various materials (e.g., glass, plastic) had been exploited to coat polished or chemically pre-treated (CT) titanium alloy surfaces. Among the different deposition techniques generally used for catechols, the dip-coating methodology was chosen as a simple and efficient strategy for surface modification to get uniform and thin organic-based films mostly devoid of surface defects.

To this aim, two titanium substrates were considered as bulk surfaces: polished (named Control) and CT Ti6Al4V alloy. These surfaces are intended mainly, but not exclusively, for orthopedic (artroprostheses or trauma devices) implants but can be further implemented on different medical devices at bone healing. The polished surface is close, for example, to the percutaneous current surface of fracture fixation devices. The CT surface is suitable for bone contact with several advantages due to its intrinsic bioactivity: formation of a surface titanium oxide layer (300–400 nm thick) with a nanoporous sponge-like morphology, high hydroxylation degree, improved adhesion of hard tissue, limited bacterial adhesion (anti-microfouling action compared to polished surfaces), low macrophage proliferation, high and selective protein adsorption (proteins are not adsorbed on the surface proportionally to the amount of each in physiological fluids, but adhesive proteins are preferentially adsorbed), improvement of thin film coating adhesion, and grafting ability, as previously demonstrated.^8,32−34^

The coatings of CA/HMDA on the titanium surfaces, characterized from the chemical and physical standpoints and their antioxidant properties and redox responsive behavior and not highlighted in the previous work,^29^ were investigated in comparison with the uncoated substrates. Moreover, the cytocompatibility and the antioxidant protective effects under pro-inflammatory conditions of the developed coatings were also evaluated toward human mesenchymal stem cells (hMSCs); finally, the coatings’ ability to prevent the surface bacterial colonization and further biofilm development was assessed toward the joint pathogen *Staphylococcus aureus*.

## 2 Materials and Methods

CA, HMDA, 2,2-diphenyl-1-picrylhydrazyl (DPPH), ferric chloride (III) hexahydrate, 2,4,6-tris(2-pyridyl)-*s*-triazine (TPTZ), Folin–Ciocalteu (F&C) reagent, gallic acid, and phosphate buffer saline (PBS) were purchased from Sigma-Aldrich (Merck, Milan, Italy) and used without any further purification.

### 2.1 Substrate Preparation and Chemical Pre-Treatment

All Ti6Al4V (ASTM B348-10, Titanium Consulting & Trading, composition: Fe 0.13, C 0.011, N 0.01, H 0.002, O 0.15, Al 6.11, V 4.12, Ti balance) samples (discs with 10 mm diameter and 2 mm thickness) were polished with SiC abrasive papers (320 and 400 grit) and subsequently washed with acetone (5 min) and ultrapure water (2 × 10 min) in an ultrasonic bath in order to remove surface contaminants. Some of these polished samples were considered as control, while a second group underwent a patented CT,^32^ which involves a first etching in diluted hydrofluoric acid (for removal of the native oxide layer) and subsequent oxidation in hydrogen peroxide (for the formation of a nanotextured oxide layer) as reported.^33^

### 2.2 General Procedure for Substrate Coating and Analysis

CA (40 mg) dissolved in the minimal amount of methanol (200 μL) was added to a 1 mM solution of HMDA (26 mg) in 0.05 M sodium carbonate buffer (final volume 220 mL) at pH 9.0 to a final concentration of 1 mM (catechol/amine molar ration of 1:1), and the mixture was left under vigorous stirring. Control and CT substrates (10 mm diameter and 2 mm thickness) were dipped into the reaction mixture and left under stirring for 24 h. The two coated samples termed Control + CA/HMDA and CT + CA/HMDA were then rinsed with distilled water in an ultrasonic bath, dried in air, and analyzed by UV–vis spectrophotometry, fluorescence microscopy, FT-IR, X-ray photoelectron spectroscopy (XPS), and zeta-potential titration.

Immediately before the dip-coating procedure, the titanium alloy surfaces were subjected to UV irradiation for 1 h (UV-C 40 W; 253.7 nm) to remove contaminations and to enhance the reactivity of the surfaces.^35^

### 2.3 UV–vis Analysis

UV–vis spectra were recorded on a Jasco V-730 spectrophotometer or Shimadzu UV2600 equipped with the integrating sphere. Experiments were performed using one sample for each type of surface.

### 2.4 Total Phenolic Content Assay

The F&C test was performed by adapting a procedure currently used to quantify the total phenolic content.^36^ Briefly, the two coated samples (Control + CA/HMDA and CT + CA/HMDA) were dipped in a solution consisting of the F&C reagent, 75 g/L Na_2_CO_3_, and distilled water in a 1:3:16 v/v/v ratio. After 2 h of incubation at room temperature, the absorbance at 760 nm was measured. Control experiments were also run on uncoated Control and CT samples. Results were expressed as gallic acid equivalents (GAE) and reported as average ± standard deviation (SD). Experiments were run using three samples for each type of surface.

### 2.5 Fluorescence Microscopy

Fluorescence microscopy observations were performed for the visualization of the CA/HMDA coatings, exploiting polyphenols’ autofluorescence.^21,37^ A confocal microscope (LSM 900, ZEISS) with 3 different filters (DAPI—blue, ALEXA FLUOR 489—green and, RHODAMINE—red) and excitation wavelength of 573 nm was used. An exposure time of 1 s and a magnification of 200× were adopted. Experiments were performed using one sample for each type of surface.

### 2.6 Topography and Roughness Measurement

Surface topography and roughness were investigated by means of confocal microscopy (LSM 900, ZEISS) using a 20× objective. The ConfoMap software was used for image elaboration and surface roughness values were obtained according to ISO 25178. Experiments were performed using one sample for each type of surface.

### 2.7 Zeta Potential Titration Curves

Zeta potential measurements were performed on bare and coated samples by means of an electrokinetic analyzer (SurPASS, Anton Paar) equipped with an adjustable gap cell. A couple of samples was fixed in the cell with surfaces to be analyzed facing each other and forming a gap, adjusted at about 100 μm. 0.001 M KCl was used as the electrolyte and 0.05 M HCl and NaOH for the titration. The measurement started at the pH of the electrolyte (5.6), and a first curve was obtained titrating the solution in the acidic range by means of the instrument’s automatic titration unit. After instrument washing, the basic curve was obtained starting again with a fresh electrolyte at pH 5.6 and titrated in the basic range. Experiments were run using one couple of samples for each type of surface.

### 2.8 Water Contact Angle Measurement

Wettability was measured by means of the sessile drop method through contact angle measurements (DSA-100, KRÜSS GmbH) with ultrapure water as the wetting fluid. A drop (5 μL) of ultrapure water was deposited on the sample surface and the angle measured with the instrument software (drop shape analysis). The surface free energy of the surfaces has been estimated by means of Neumann’s equation.^38^ Experiments were run using three samples for each type of surface, and the results are reported as average ± SD.

### 2.9 Adhesion Tape Test

Coating adhesion was evaluated by means of the tape adhesion test according to the ASTM D 3359 standard.^39^ A grid of parallel cuts was prepared on the coated surface by means of a cutter, and a standard adhesive was applied to the surface and then removed at once. The test was performed on one sample per type of surface. The surface was then inspected visually and by means of scanning electron microscopy equipped with energy-dispersive spectroscopy (SEM–EDS, JEOL, JCM 6000 plus and JED 2300) for localized analyses of the chemical composition in specific areas to understand coating permanence/detachment. The entity of coating detachment was estimated according to the ASTM D 3359 standard.

### 2.10 X-ray Photoelectron Spectroscopy

The chemical composition of the outermost surface layer as well as the functional groups exposed on the surface were analyzed by means of XPS (PHI 5000 VERSAPROBE, PHYSICAL ELECTRONICS). Both survey spectra (0–1200 eV) for individuation and quantification of chemical elements and high-resolution ones (C, O, and N regions) for the detection of functional groups were acquired. In order to guarantee the charging effect compensation, spectra were referenced by setting the hydrocarbon C 1s peak to 284.80 eV. Experiments were performed using one sample for each type of surface.

### 2.11 Fourier-Transform Infrared Spectroscopy

FT-IR analyses of the coated surfaces (one sample per type of surface) were done in the ATR mode using a Thermo Fisher Nicolet 5700 spectrophotometer equipped with a Smart Performer accessory mounting a ZnSe crystal for the analysis of solid samples. Experiments were performed using one sample for each type of surface.

### 2.12 Antioxidant Properties

#### 2.12.1 DPPH Assay

The assay was performed as previously described with slight modifications.^40,41^ Control + CA/HMDA- and CT + CA/HMDA-coated substrates were immersed in a solution of 50 μM DPPH in ethanol (2 mL), and the antioxidant power was evaluated by UV–vis spectroscopy measuring the absorbance of the solution at 515 nm over time up to 15 days.

In other experiments, after 15 days of dipping in the DPPH solution, Control + CA/HMDA- and CT + CA/HMDA-coated substrates were rinsed with distilled water, air dried, and immersed in a freshly prepared DPPH solution for other 2 weeks and then treated as above. The absorbance at 515 nm was monitored over time up to 56 days.

In control experiments, the DPPH assay was run on the pristine Control and CT materials.

In additional experiments, Control + CA/HMDA- and CT + CA/HMDA-coated substrates (two samples for each type of surface) were immersed in 50 μM ethanolic solution of DPPH (2 mL), and the antioxidant power was evaluated as described above, measuring the absorbance over time up to 72 h.

Values are expressed as DPPH decay over time. Experiments were run using three samples for each type of surface.

#### 2.12.2 Ferric Reducing/Antioxidant Power Assay

The assay was performed as previously described with slight modifications.^42^ Briefly, Control + CA/HMDA- and CT + CA/HMDA-coated substrates were immersed into a solution of the ferric reducing antioxidant power (FRAP) reagent (2 mL), prepared by sequentially mixing 0.3 M acetate buffer (pH = 3.6), 10 mM TPTZ in 40 mM HCl, and 20 mM ferric chloride in water (10:1:1 v/v/v ratio). The absorbance of the solution at 593 nm was measured over time up to 7 days. Values are expressed as absorbance of the Fe^2+^–TPTZ complex over time. In control experiments, the FRAP assay was run on the pristine Control and CT materials. In other experiments, after 7 days of dipping in the FRAP solution, Control + CA/HMDA- and CT + CA/HMDA-coated substrates were rinsed with distilled water, air dried, and immersed in a freshly prepared FRAP solution for another week and then treated as above. The absorbance at 593 nm was monitored every 7 days up to 56 days. Experiments were run using three samples for each type of surface.

### 2.13 Kinetics of CA/HMDA Release from the Titanium Alloys

The kinetics of release of CA/HMDA compounds was evaluated by dipping the coated substrates in 15 mL of PBS 1× at pH 7.4 at 37 °C for 28 days. The medium was repeatedly refreshed at 1, 7, 14, and 28 days. At each experimental time, the TPC assay was performed by adding 2 mL of the medium that has been in contact with the sample to a solution of the F&C reagent, 75 g/L Na_2_CO_3_, and water prepared as described above (final volume 8 mL). After 2 h of incubation at 25 °C, the absorbance at 760 nm was measured by UV–vis spectroscopy. After 4 weeks, the TPC assay was performed also on the coated substrates (Control + CA/HMDA and CT + CA/HMDA) using the above-described protocol. Results were expressed as GAE and all the experiments were run using three samples for each type of surface.

### 2.14 *In Vitro* Cytocompatibility Evaluation

Commercially available hMSCs (C-12974 from PromoCell, Heidelberg, Germany) were used for cytocompatibility evaluation as representative cells for the self-healing process. Cells were cultivated with low-glucose DMEM (Sigma-Aldrich) supplemented with 15% fetal bovine serum (FBS, Sigma-Aldrich) and 1% antibiotics at 37 °C and a 5% CO_2_ atmosphere. Cells were cultivated until 80–90% confluence, detached by a trypsin–EDTA solution (0.25% in PBS), harvested, and used for experiments. Cells were directly dropwise (100 μL/specimen) seeded onto the surface of the specimens in a defined number (2 × 10^4^ cells/specimen) for 4 h to allow adhesion; afterward, specimens were submerged with 1 mL of fresh medium and cultivated for 24–48–72 h. At each time-point, the viability of the adhered cells was evaluated by means of their metabolic activity by the colorimetric metabolic assay alamar blue (alamarBlue, ready-to-use solution from Life Technologies) by directly adding the dye solution (0.0015% in PBS) in the medium. After 4 h of incubation in the dark, the fluorescent signal (expressed as relative fluorescent units—RFU) was detected at 590 nm by a spectrophotometer (Spark, from Tecan, Switzerland). Experiments were performed in triplicate.

Moreover, at the last 72 h time-point, the fluorescent live/dead assay was applied to visually check for viable cells (live/dead, viability/cytotoxicity kit for mammalian cells from Invitrogen); images were collected with a digital EVOS FLoid microscope (from Life Technologies).

Finally, the morphology of cells grown onto control and coated specimens was visually investigated by SEM imaging; briefly, specimens were fixed with 4% glutaraldehyde (20 min, room temperature), dehydrated by the alcohol scale (70–80–90–100%, 2 h each), treated with hexamethyldisilazane (5 min, 2×), and cover-sputtered with gold. Images were collected with a JEOL IT500 scanning electron microscope at various magnifications.

Experiments were performed using 3 replicates of each sample at each time-point for all the described assays.

### 2.15 Scavenging Activity under the Pro-Inflammatory Condition

The anti-inflammatory activity of the specimen was evaluated by simulating a pro-inflammatory condition *in vitro*.^43^ Briefly, hMSCs were seeded in a defined number (2 × 10^4^ cells/well) into 24 multiwell plates and allowed to grow for 24 h; then, 300 mM hydrogen peroxide^44^ was introduced in the medium and left to act for 3 h. Afterward, the fluorescent dye CellROX (from Thermo Fisher Scientific) was applied to stain the cells that have incorporated oxygenated species. Cells were counter-stained with 4′,6-diamidino-2-phenylindole (DAPI) and phalloidin (both from Thermo Fisher Scientific) to visualize nuclei and cytoskeleton F-actin filaments, respectively. Images were collected by confocal microscopy (Leica TCS SP8 LIGHTNING confocal laser scanning microscope). Experiments were performed using 3 replicates of each sample.

### 2.16 Antibacterial Activity Evaluation

A multi-drug resistant (MDR) strong biofilm-former commercial strain of the joint pathogen *S. aureus* (*S. aureus*, ATCC 43300) was purchased from the American Type Culture Collection (ATCC, Manassas, USA) and cultivated following the manufacturer’s instructions. Briefly, bacteria were cultivated in trypticase soy agar plates (Sigma-Aldrich) and incubated at 37 °C until single colonies were formed; then, 2 colonies were collected and diluted in 20 mL of Luria Bertani broth (Sigma-Aldrich) and incubated overnight under agitation (90 rpm). A fresh broth culture was prepared prior to experiments by diluting the overnight broth culture with fresh medium till a final concentration of 1 × 10^5^ cells/mL corresponding to an optical density of 0.001 at a 600 nm wavelength (measured by a spectrophotometer, Spark, from Tecan, Switzerland).

Specimens were directly infected by 1 mL of the above-mentioned bacterial suspension and incubated for 24 h; then, the metabolic activity of the adhered bacteria was evaluated by the colorimetric metabolic assay alamar blue as detailed earlier, whereas the fluorescent live/dead assay (BacLight, bacterial viability kit for microscopy, Invitrogen) was used to visually detect viable adhered colonies; images were collected by confocal microscopy (Leica TCS SP8 confocal laser scanning microscope, Leica Microsystems). Experiments were performed in triplicate.

Finally, to assess the thickness and the distribution of the 3D biofilm-like bacterial structures formed on the control and test specimens’ surface, SEM images were collected as detailed earlier and analyzed using the SMILE VIEW map software (JEOL, Tokyo, Japan).^45^

Experiments were performed using 3 replicates of each specimen at each time-point for all the described assays.

### 2.17 Statistical Analysis of Data

Experiments aimed at the biological evaluation of the specimens were performed using 3 replicates for each assay and each time-point. Results were statistically analyzed using SPSS software (v. 20.0, IBM, USA). Groups were compared by one-way ANOVA using Tukey’s test as the post-hoc analysis. Significant differences were established at *p* < 0.05.

## 3 Results and Discussion

**Figure 1:**
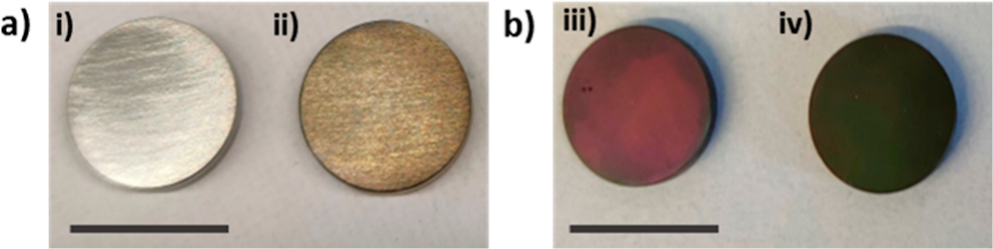
Digital pictures of (a) polished titanium surfaces before (i) and after CA/HMDA coating (Control + CA/HMDA) (ii) and (b) CT titanium surfaces before (iii) and after CA/HMDA coating (CT + CA/HMDA) (iv). Bar scale = 1 cm.

The HMDA-modified dip-coating protocol previously reported for glass substrates^29^ was applied to titanium alloy samples. The control sample appears shiny gray, as typical for coarsely polished titanium, while the chemically treated sample is reddish due to the presence of a nanotextured titanium oxide layer of about 400 nm in thickness. Immersion of both polished (Control) and CT samples into a solution containing 1 mM CA at pH 9.0 in the presence of HMDA (1 mM) resulted in the deposition after 24 h of benzacridine-based films characterized by yellowish (Figure 1a) or greenish (Figure 1b) colors, respectively, on control (Control + CA/HMDA) and CT (CT + CA/HMDA) substrates that are well detectable by visual inspection.

To confirm the presence of coated polyphenols on the solid samples, UV–vis analyses were performed in reflectance modality on both the coated samples and bare ones for comparison (Figure S1).

The CT substrate had a surface oxide layer (about 400 nm thick),^46^ which was transparent to visible light, causing a reduction of reflectance with respect to the polished surface (Control) and multiple reflections at the interface with the metallic substrate, which resulted in typical ripples in the UV spectrum. The amplitude of these ripples slightly decreased after the CA/HMDA coating as a confirmation of the presence of a further non-reflective continuous film due to the organic coating. The polished control (Control) showed a reflectance spectrum typical of the native titanium oxide.^47^ The surface reflectance was significantly reduced after the CA/HMDA coating, especially at low wavelengths (UV region), suggesting the presence of a non-reflective organic coating.

The amount of phenolic coating deposited on the substrates was evaluated by means of the F&C test adapted to solid samples. As expected, the GAE values resulted in a negligible change on the pristine titanium surfaces (Control and CT), while they increased appreciably after the dip-coating procedure of 1.6 ± 1.8 × 10^–3^ and 1.2 ± 2.5 × 10^–3^ μg/mL for the CT + CA/HMDA and Control + CA/HMDA, respectively, thus confirming the effectiveness of the adopted coating procedure.

### 3.1 Morphological Characterization of the Coated Surfaces

**Figure 2:**
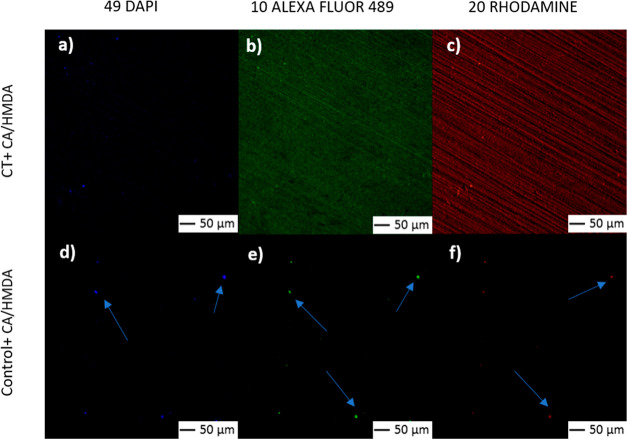
Fluorescence microscopy images of CT + CA/HMDA (a–c) and Control + CA/HMDA surfaces (d–f). Arrows indicate small agglomerates visible on Control + CA/HMDA (d–f).

In other experiments, the presence and distribution of the organic coating were also evaluated by means of fluorescence microscopy (Figure 2).

Figure 2 shows the fluorescence microscopy images acquired with different filters on the titanium samples. In particular, the coating appeared continuous and homogeneous on the CT samples (CT + CA/HMDA), as evidenced by Figure 2a−c, whereas fluorescent small agglomerates (6–8 μm) were visible on Control + CA/HMDA (Figure 2d–f). Taking into account these observations, a thinner layer can be supposed to be deposited on the control surface with respect to the one deposited on the CT sample.

**Figure 3:**
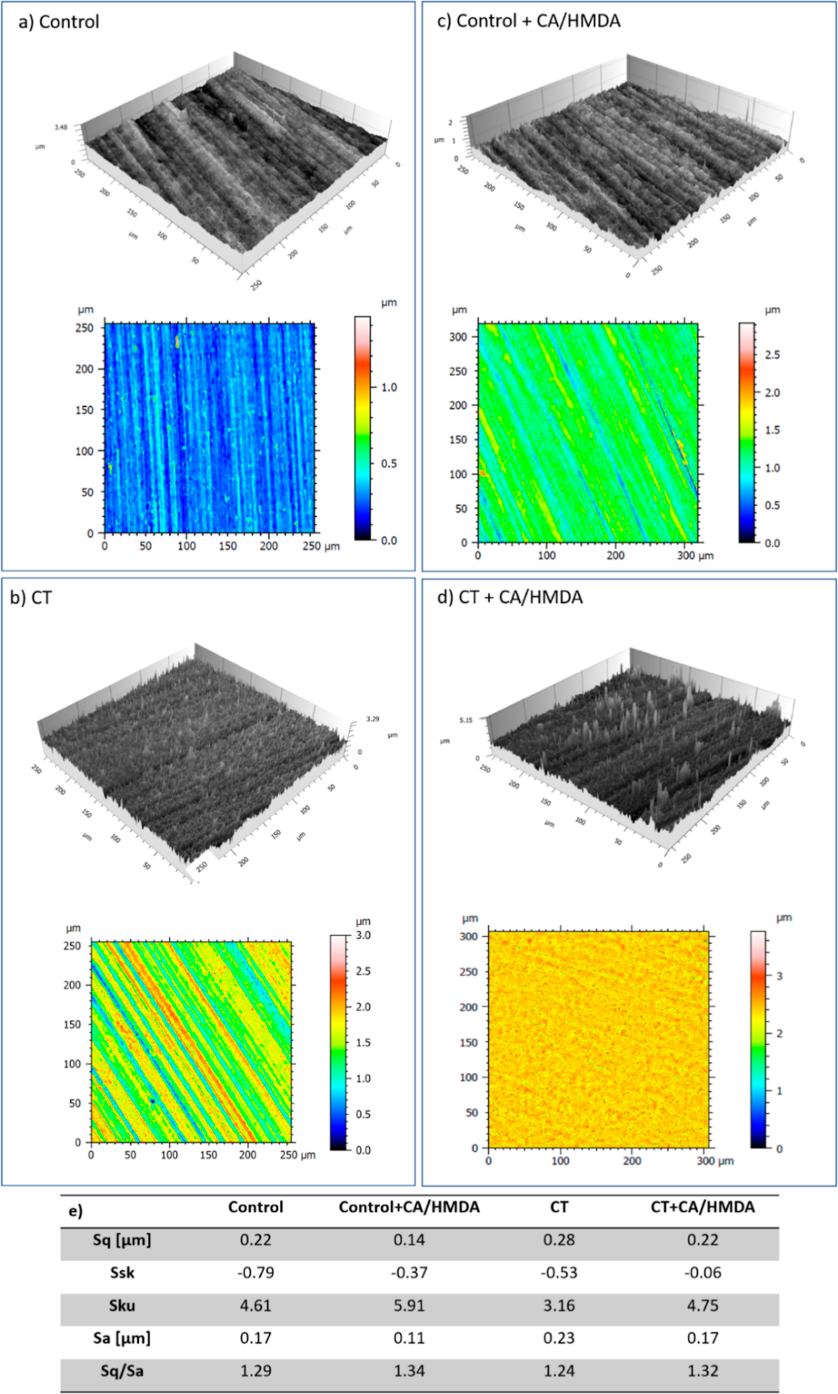
Confocal microscopy images of the tested surfaces (a–d) and roughness measurements according to ISO 25178 (e).

Further insight into the surface topography and roughness of the coated samples was obtained by means of confocal microscopy (Figure 3).

The polishing tracks were clearly visible on both substrates before the coating (Figure 3a,b) due to the coarse polishing procedure (SiC abrasive papers up to 400 grit). As noticeable in Figure 3c,d, these tracks were less apparent after the coating, especially on the CT + CA/HMDA sample.

Noteworthily, all the average roughness values were low and close to 0.2 μm, a value that corresponds to the threshold generally reported in the literature not to increase bacterial adhesion but suitable for osseointegration.^48^ Such low roughness values were not expected to involve any significant difference in the surface area among the samples.^48^

Although the typical CT nanotexture cannot be observed at this low magnification, it was pointed out by the higher roughness (Sa) value with respect to the control, as already reported.^49^ The average surface roughness (Sa) slightly decreased after the coating (CT + CA/HMDA *vs* CT, and Control + CA/HMDA *vs* Control) as a confirmation of the presence of a homogeneous organic layer on the surfaces, which attenuated surface asperities.

Skewness (Ssk) values were negative and close to 0, evidencing that there was no strong prevalence of peaks or valleys, but they were almost in a balanced proportion, with a small prevalence of negative features, on all surfaces, as expected because of the subtractive surface treatments (grinding and etching). After the coatings, the negative values were slightly decreased in both cases, with the CT + CA/HMDA sample showing the larger reduction and evidencing thus the presence of a thicker coating able to better cover the surface features. These results are of great interest considering that a low Ssk value is reported to have a positive effect on the biological response of osteoblasts and mesenchymal cells.^50^

Moreover, the CT sample showed a kurtosis (Sku) value (a parameter that describes the peakedness of a surface) close to 3 and an Sq/Sa ratio close to 1.25: both these parameters were in line with an almost Gaussian height distribution of the chemically etched sample. The higher value of Sku (>3) and Sq/Sa ratio (>1.25) of the control sample was typical of a ground surface with sharp peaks. The presence of sharp peaks is also evidenced by Sku and the Sq/Sa ratio on both CT + CA/HMDA (4.95) and Control + CA/HMDA (5.91): this is an unexpected morphological defect of the coatings, visible especially in Figure 3d. A Sku value close to 3 is reported to have a positive effect on the biological response of osteoblasts and mesenchymal cells, which easily colonize surfaces with a smooth Gaussian morphology.^50^

### 3.2 Zeta Potential Titration Measurements

**Figure 4:**
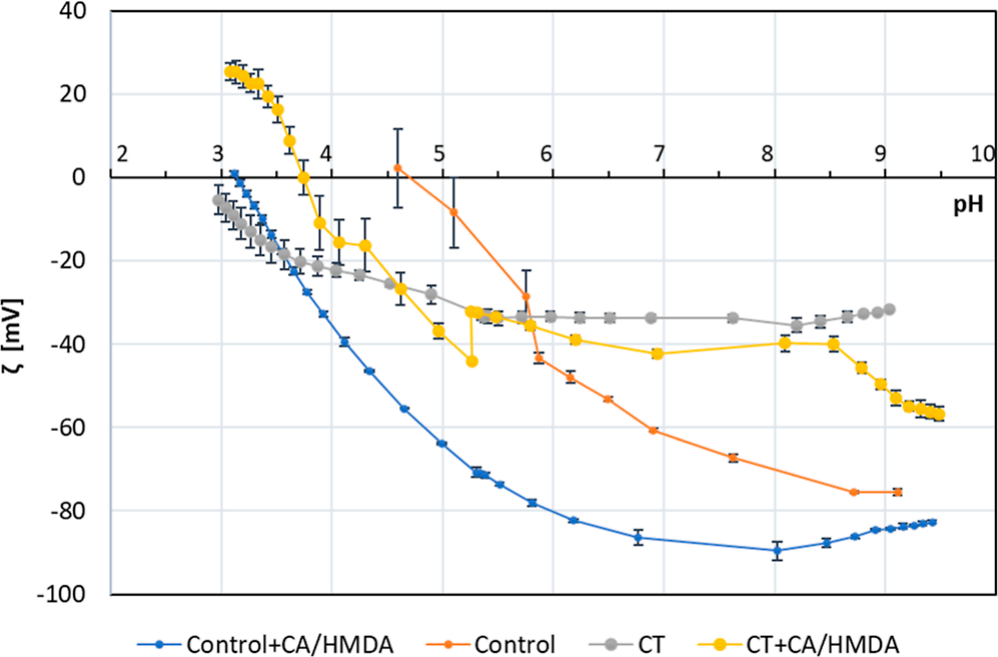
Zeta potential titration curves of the reference and coated samples. Each measurement has been performed on a couple of samples and repeated 4 times for each pH value.

In order to investigate the surface zeta potential as a function of pH and to estimate the coating chemical stability/reactivity in liquid environments at different pH, zeta potential electrokinetic measurements were performed on the bare and coated surfaces. Figure 4 reports the zeta potential titration curves of the tested surfaces.

The control sample had an isoelectric point of 4.7, a curve without an evident plateau (both features attributable to the absence of acidic/basic functional groups), and a quite high SD in the acidic range (index of incipient corrosion of the surface and high surface conductivity), as expected for a polished titanium surface.^51^ After the deposition of the CA/HMDA coating (Control + CA/HMDA), an acidic shift of the isoelectric point could be observed (down to 3.14), indicating the prevalence of acidic functional groups such as the hydroxyl and carboxylic groups of the trioxybenzacridinium moieties. This is confirmed by a plateau in the basic range with onset at pH 7.5 which indicated that all the acidic groups are deprotonated at this pH. Moreover, the SD was extremely low at all pHs, indicating the increased chemical stability of the coating in the explored pH range. As previously observed by the authors,^52^ the SD of zeta potential titration curves is an estimation of surface stability/reactivity at different pHs. If the surface changes during the measurement (due to the reaction with the electrolyte), the SD is high; on the other hand, if the surface is stable, the SD is low. The change of the isoelectric point, shape of the curve, and SDs observed after the coating confirmed the presence of a continuous and chemically stable organic coating on the polished titanium surface.

The CT sample showed a significant acidic shift of the isoelectric point, compared to the Control, attributable to the presence of acidic functional groups, as confirmed by the pronounced plateau in the basic pH region, with onset at pH 5.5, at which the groups are completely deprotonated, as previously reported by the authors:^51^ these functional groups are the −OH groups exposed by the oxide layer formed during the CT. The SD is small for all points, evidencing a corrosion protection ability of the oxide layer in the whole pH range. Finally, the titration curve of CT had a lower slope around the isoelectric point with respect to the control polished surface because of the larger hydrophilicity of this substrate, rich in hydroxyl groups, and the lower adsorption of charged ions from the solution in replacement of water. After the coating (Figure 4—CT + CA/HMDA), a basic shift of the isoelectric point up to 3.7 could be observed together with the presence of two small plateaus at 4.0 and 5.5. The shift of the isoelectric point toward a higher value evidenced the prevalence of functional groups with a basic character (such as amino groups of HMDA), denoting extensive incorporation of diamine during CA polymerization.^29^ The presence of two plateaux could be associated with the presence of two functional groups with different pKa. An increase in the SD in the acidic range (from pH 4.0 down to pH 3.0) and a “jump” of the zeta potential at pH 5.6 between the acid (tested as first) and the basic curves evidenced a significant reactivity of the surface at acidic pH. The “jump” is expected when a surface is reactive in the acidic range and the titration is made on the same set of samples in the two ranges of pH (as it usually occurs).

The modifications of the zeta potential curves after coating confirmed the presence of the CA/HMDA coating on both the substrates. Although the autoxidation of CA in the presence of HMDA produces trioxybenzacridinium moieties bearing carboxyl and amine functionalities, the differences in the isoelectric points and in the reactivity of the coated samples suggested a different distribution of the functional groups on the outer surface depending on the titanium substrate. In particular, the coating exposed, in the outermost layer in contact with the liquid environment, mainly the OH groups of polyphenols when it was deposited on the control polished substrate, while it was much more reactive in the acidic range and it exposed a higher number of basic functional groups when it was deposited on the CT substrate.

### 3.3 Surface Wettability

**Figure 5:**
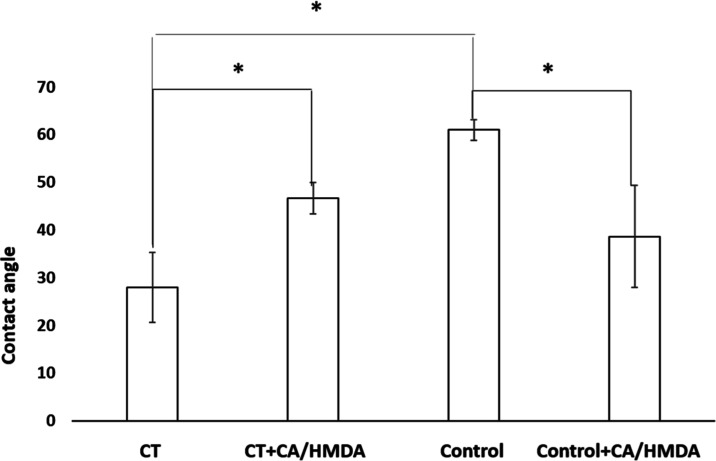
Contact angle measurements on the samples before and after the coating (* = *p* < 0.05, one-way ANOVA). Reported are the mean ± SD values of three samples.

The results of the water contact angle measurements are reported in Figure 5.

CT showed a significantly lower contact angle than the polished substrate due to a high amount of hydroxyl groups exposed on the surface oxide layer, in agreement with the zeta potential titration curve.^8,33^ After the coating, the contact angle value of CT increased. The value of the contact angle (close to 40°) was similar for the two surfaces and significantly different from the uncoated ones. This would suggest a moderately hydrophilic nature of the CA/HMDA coating that prevailed over the character of the underlying titanium surface.

An estimation of the surface free energy of the different surfaces was performed by applying Neumann’s equation.^38^ Even if this model is not as reliable as that of the Owens–Wendt is, it gave reasonable estimations and it returned results in agreement with what was already achieved by using different liquids (e.g., water and hexadecane) on the CT and control samples.^53^ The CT sample had the highest values of surface energy (65 mJ/m^2^), followed by the two coated samples (51–57 mJ/m^2^), and the Control sample had the lowest value (48 mJ/m^2^). Noteworthily, all the surfaces had surface energy values above the threshold of 41 mJ/m^2^, which is generally considered needed for cell adhesion.^53^

Surface wettability measured by the contact angle method includes the effect of both surface chemistry and topography. An estimation of the surface wettability only related to the surface chemistry can be obtained by the slope of the zeta potential titration curve around the isoelectric point (Figure 4). The slope of the CT sample was significantly lower than the one of the control sample, further confirming its higher wettability. The slope of the curve was almost the same for coated surfaces, confirming their good and analogous wettability. These observations thus suggested that wettability was mainly affected by surface chemistry for the here-analyzed samples.

### 3.4 Coating Mechanical Adhesion Strength

**Figure 6:**
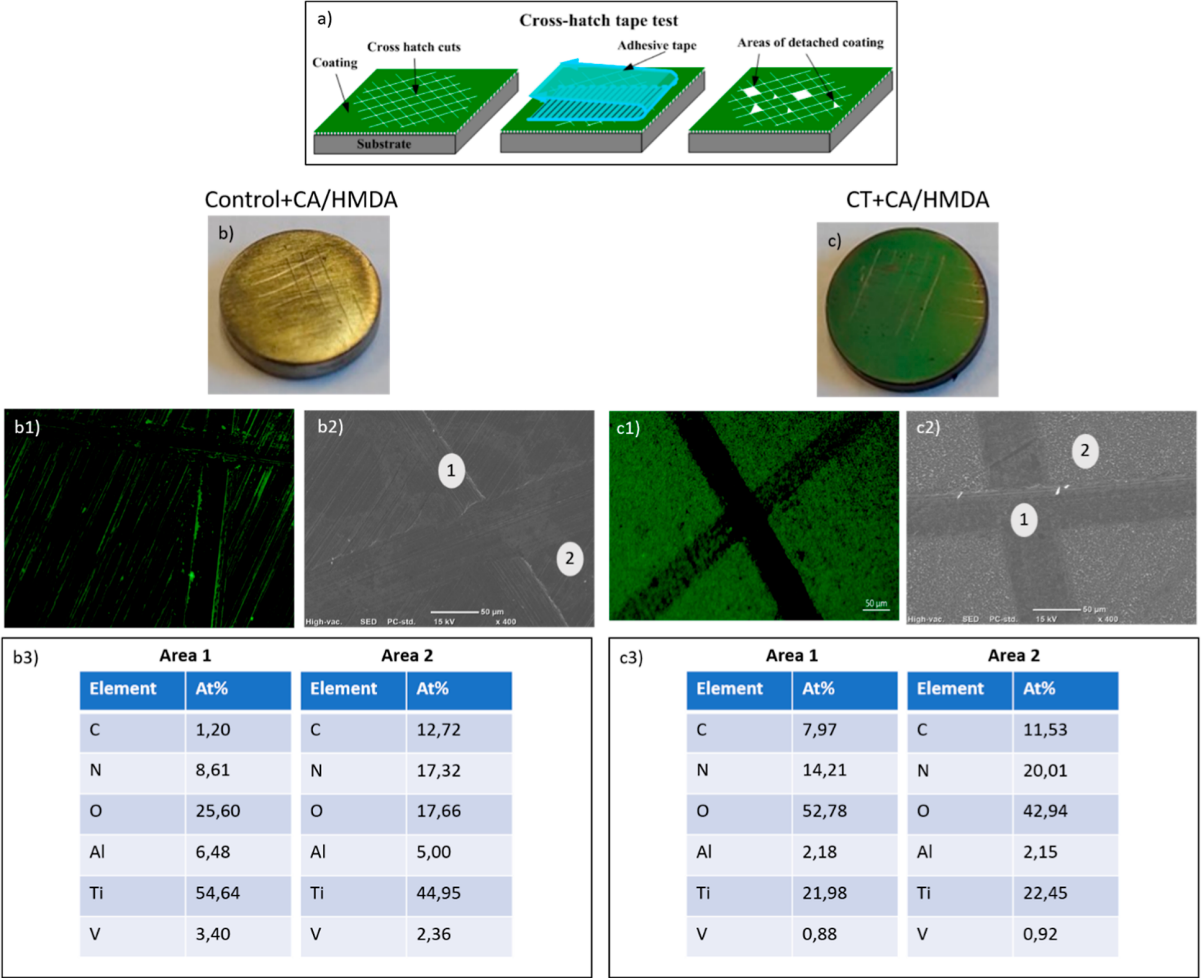
Tape adhesion test: (a) schematic representation of the test. (b) Macro image of the Control + CA/HMDA sample after the tape test. (b1) Fluorescence image of the Control + CA/HMDA sample after the tape test. (b2) SEM image of the Control + CA/HMDA sample after the tape test. (b3) EDS analyses on the selected zones [areas 1 and 2 in (b2)]. (c) Macro image of CT + CA/HMDA after the tape test .(c1) Fluorescence image of CT + CA/HMDA after the tape test. (c2) SEM image of CT + CA/HMDA after the tape test. (c3) EDS analyses on the selected zones [areas 1 and 2 in (c2)].

The coating adhesion was evaluated by means of the tape adhesion test (ASTM D 3359). As shown in Figure 6, the macro appearance of the surfaces was almost unaltered after the test (only the grid is visible but no color variations are noticeable); moreover, the fluorescence microscopy images of the two coated samples after the tape test confirmed the results described above. A homogeneous coating was present on the CT substrate, while it was less evident on the polished one. Furthermore, the coating was maintained after the test, except in the cross-cuts, on both substrates. SEM–EDS analyses further confirmed the presence of the coating after the tape test outside the cuts by means of a high value of carbon and nitrogen mainly due to the organic coating and a reduced Ti, Al, and V content coming from the inorganic support. According to the ASTM D 3359 standard, the coating adhesion on both substrates can be classified as 5B, which is optimal adhesion.

### 3.5 Chemical Composition of the CA/HMDA Coatings

FT-IR spectra taken in the ATR mode on Control + CA/HMDA and CT + CA/HMDA showed the presence of peaks attributable to benzacridine derivatives (Figure S2). In particular, the spectra were dominated by the intense peaks at 2934 and 2854 cm^–1^, which are representative of the asymmetrical and symmetrical vibrations of CH_2_, while the well-defined band at 1745 cm^–1^ would be evidence for the presence of carboxyl groups. The broad band around 3500 cm^–1^ arises from the phenol and carboxyl OH groups. Weak but well-detectable peaks are observed at 3314, 1240, and 1160 cm^–1^ attributable to NH stretching vibration, C–N bond stretching vibration, and stretching of the C–O bonds in the phenolic groups, respectively.

A deeper insight into the chemical composition of the coated surfaces was got through XPS analyses (Table 1).

**Table 1:** Chemical Composition of the Different Samples from XPS Survey Analyses (at %)

element [at %]^a^	control	control + CA/HMDA	CT	CT + CA/HMDA
C	46.9	76.5	12.1	70.6
O	38.8	14.7	62.9	19.2
N		5.8		8.7
Na		2.1		0.8
Si		0.7	1.6	
Cl		0.3		
Ca				0.4
Ti	12.3	0	21.0	0.4
Al			2.4	

a Values were obtained on one sample per type, an error range of ±5–15% from the atomic concentration of each element.

A significant increase in the surface carbon (and nitrogen) content, together with a reduction of those of oxygen and titanium, could be observed on the coated surfaces compared to the substrates as a confirmation of the presence of an organic coating. Considering that Ti was not detected on both CT + CA/HMDA and Control + CA/HMDA, it could be concluded that a coating thicker than the XPS sampling depth (5–10 nm) was present in both cases. Notably, a nitrogen signal was evident only in the coated samples, as expected based on the use of HMDA as a crosslinking agent. The content of N was higher on CT + CA/HMDA than on Control + CA/HMDA: this agreed with the evidence of basic functional groups exposed by the former in the zeta potential titration curves.

**Figure 7:**
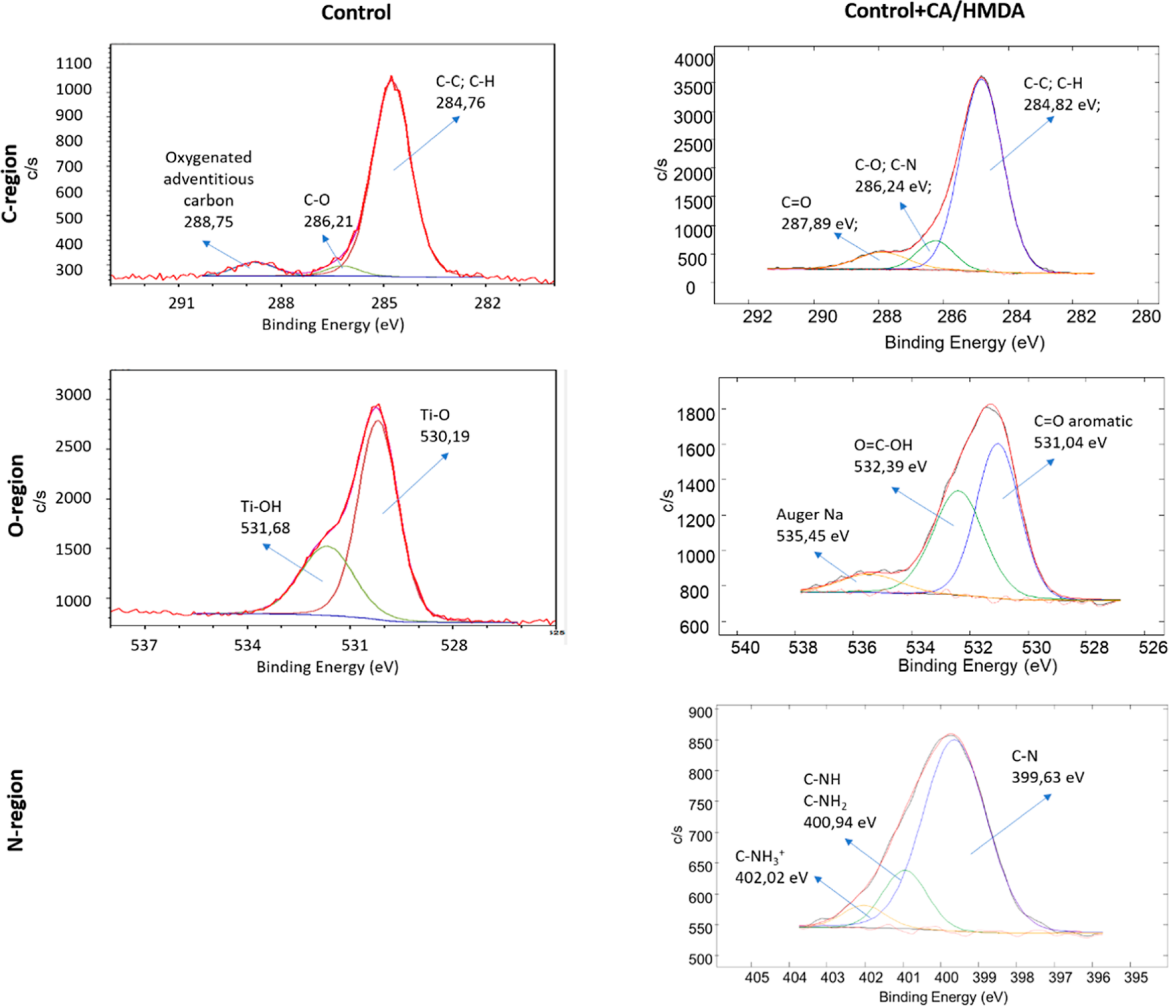
XPS high-resolution spectra of the carbon, oxygen, and nitrogen (only for the coated sample) regions for the Control and Control + CA/HMDA samples.

**Figure 8:**
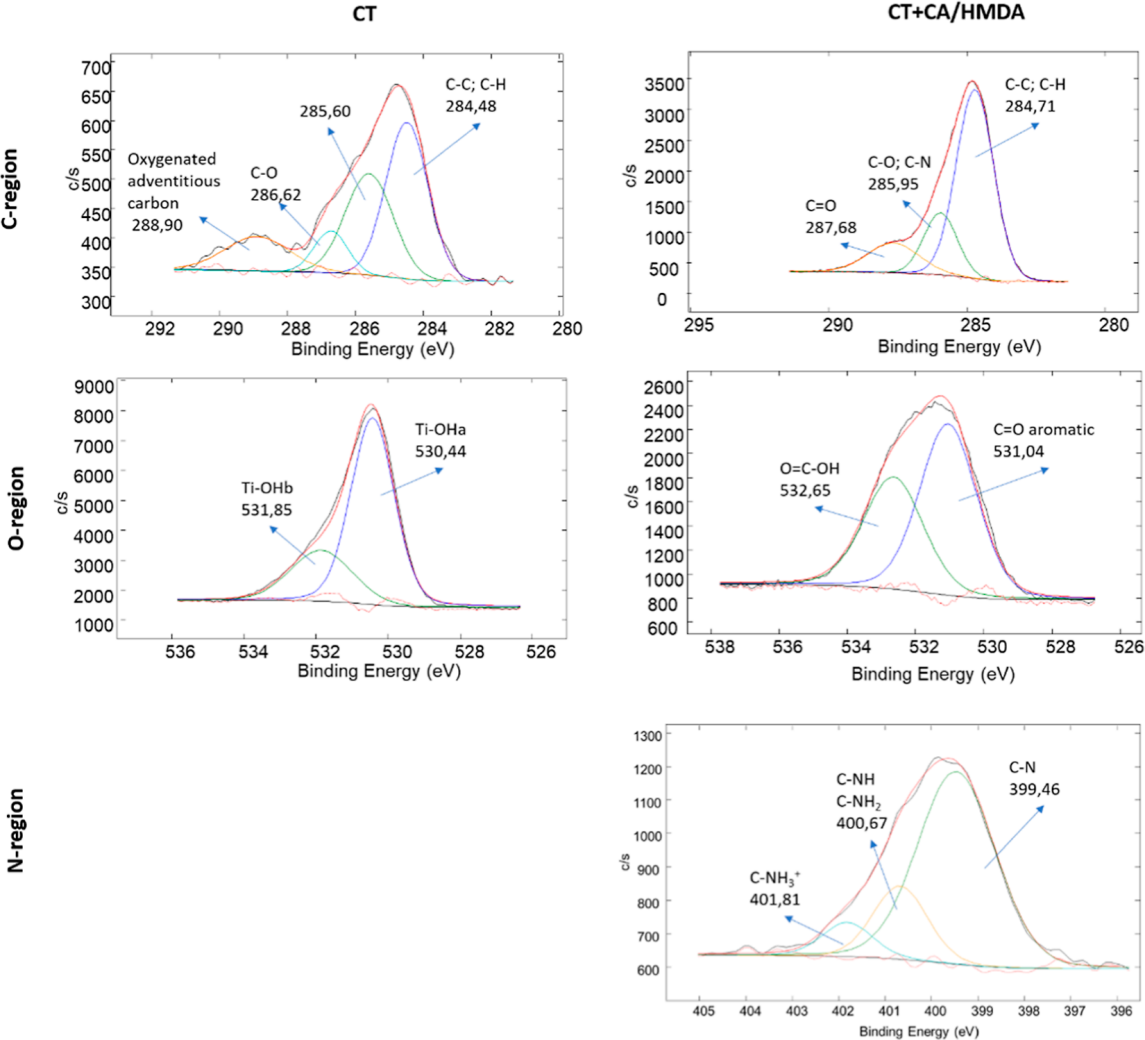
XPS high-resolution spectra of the carbon, oxygen, and nitrogen (only for the coated samples) regions for the CT and CT + CA/HMDA samples.

The high-resolution spectra of the carbon, oxygen, and nitrogen regions are reported in Figure 7 for the Control and Control + CA/HMDA and in Figure 8 for CT and CT + CA/HMDA respectively.

The high-resolution spectrum of the carbon region for the control sample (Figure 7) showed a main peak at 284.76 eV attributable to C–C and C–H from hydrocarbon contaminants from the atmosphere, always present on titanium surfaces,^54^ together with small signals at about 286.21 and 288.75 eV (C–O and oxygenated adventitious carbon, respectively), which could also be associated with surface contaminations.^21^ After the coating, the signal of carbonates disappeared, the signal at 286 eV, attributable to C–O and C–N bonds,^55^ increased, and a signal at 287.89 eV, attributable to C=O bonds,^55^ appeared; these two signals, together with the one at 284.82 eV, could be associated with the benzacridine carbonyls and more generally to the presence of the diamine crosslinked polyphenolic coating.

The high-resolution spectrum of the oxygen region for the control sample showed two main contributions at 530.19 and 531.68 eV, which can be attributed to Ti–O and Ti–OH (acid) bonds.^46^ Putting together the information from the zeta potential titration curves and XPS analysis, it could be concluded that the control substrate exposed hydroxyl groups, but they had not a strong acid/basic behavior. After the coating, the spectrum completely changed, evidencing a contribution at 531.04 eV attributable to aromatic C=O,^56,57^ a contribution at 532.39 attributable to O=C–OH,^21^ and a small contribution at 535.45 attributable to the Na auger signal.^56,57^

The high-resolution spectrum of the nitrogen region, reported only for the coated sample (Control + CA/HMDA), showed two contributions at 399.63 eV (78%) and 400.94 eV (16%), attributable to C–N and C–NH/C–NH_2_, respectively,^55,58^ and a contribution at 402.02 eV (6%) that could be attributed to C–NH_3_^+^.^59^ These again confirmed the presence of nitrogen belonging to heterocyclic aromatic rings as well as to HMDA amino groups linked to the CA-derived moiety.

The high-resolution spectra of the carbon region for the CT and CT + CA/HMDA samples (Figure 8) were analogous to the ones previously discussed, respectively, for the Control and Control + CA/HMDA samples. The only difference was that the C–N signal was higher on CT + CA/HMDA than on Control + CA/HMDA in agreement with a higher N content on this sample.

Actually, also the high-resolution spectrum of the oxygen region for the CT sample was significantly different from that of the Control due to the presence of a titanium oxide layer (400 nm thick) rich in hydroxyl groups.^46^ Two signals related to acid and basic hydroxyl groups, respectively, at 530.44 and 531.85 eV, could be clearly observed. After the coating, the spectrum of CT + CA/HMDA was similar to the one of Control + CA/HMDA: two contributions, one at 531.04 eV attributable to aromatic C=O,^56^ and one at 532.65 attributable to O=C–OH,^21^ could be observed. The sodium content on CT + CA/HMDA was lower than the one on Control + CA/HMDA and the auger signal of sodium was not visible in this case.

The high-resolution spectrum of the nitrogen region, reported only for the coated CT + CA/HMDA sample, showed two contributions at 399.46 eV (72%) and 400.67 eV (19%) attributable to C–N and C–HN/C–NH_2_, respectively.^55,58^ A contribution at 401.81 eV (9%) due to C–NH_3_^+^ was also present.^59^ These contributions were the same as the ones previously observed on Control + CA/HMDA, but in this case, there was an increase in the C–NH/C–NH_2_/C–NH_3_^+^ contributions. Considering both the zeta potential titration curves and XPS analyses, it can be concluded that the coating composition was sensitive to the chemical nature of the substrate where it was formed: when the coating was formed on the CT substrate, there was a larger number of free amino groups exposed, which accounted for a stronger basic behavior. These may be partially protonated or given the presence of carboxyl groups, formation of zwitterionic forms should be considered. Overall, this conclusion agreed with a prevalence of aliphatic amines for the coating on CT (right formula in Scheme 1) and heterocycles on the Control substrate (left formula in Scheme 1).

### 3.6 Evaluation of the Antioxidant Properties of the Coated Substrates

The antioxidant properties of the CA/HMDA films formed on the titanium surfaces were investigated through two widely adopted hydrogen atom transfer and/or electron transfer assays that are the DPPH and FRAP assays.

**Figure 9:**
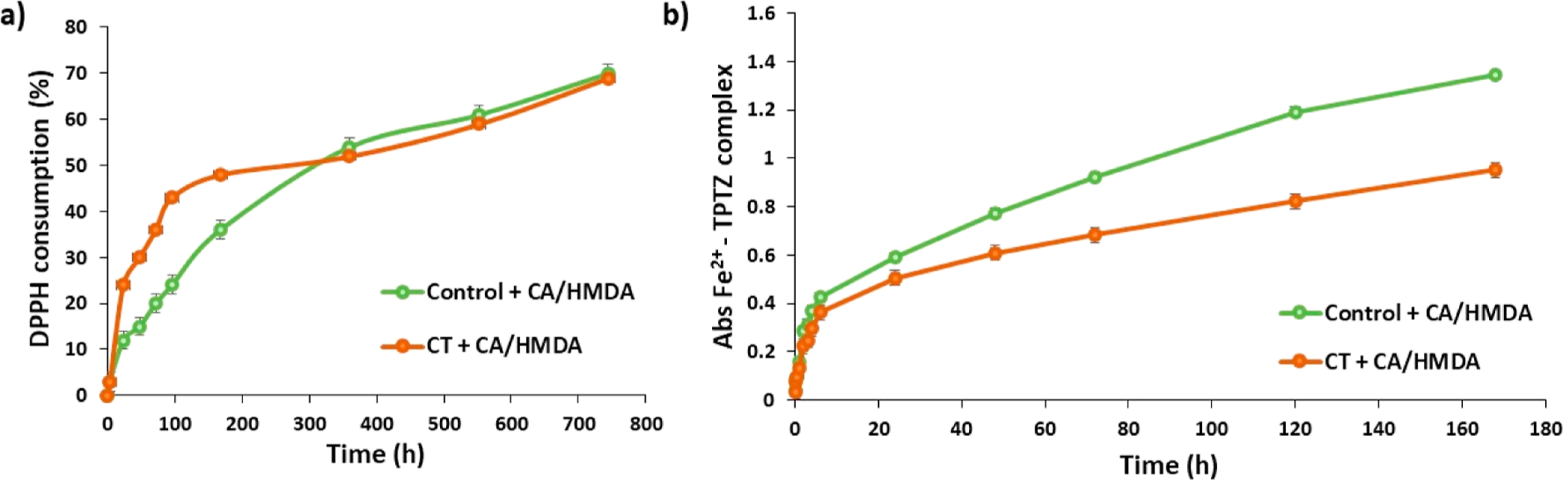
(A) DPPH reduction properties of Control + CA/HMDA and CT + CA/HMDA. (B) Absorbance at 593 nm due to the Fe^2+^–TPTZ complex induced by the coated substrates. Reported are the mean ± SD values of three experiments.

In the first case, the assay was run by dipping the coated substrates into an ethanolic solution of DPPH. Aliquots of the medium were then withdrawn periodically and analyzed spectrophotometrically. DPPH decay increased over time with both kinds of coated surfaces, reaching a 70% reduction after 29 days (Figure 9a). The CT + CA/HMDA coating showed more efficient antioxidant properties, leading to a 24% reduction of DPPH already in the first 24 h. In addition, when two coated substrates for each type of surface were dipped in the DPPH solution, well detectable effects of the coatings were observed in both cases in the first 48 h, reaching a *ca.* 64 and 70% reduction after 72 h for Control + CA/HMDA and CT + CA/HMDA, respectively (Figure S3). These results have prompted that the higher the surface area coated, the higher will be the antioxidant activity.

A similar procedure was also followed to evaluate the ferric reducing antioxidant power of the medium in which the coated surfaces were dipped for 7 days. Also, in this case, the coated substrates proved active with a well-appreciable development of the absorption Fe^2+^–TPTZ complex at 593 nm (Figure 9b), particularly for the Control + CA/HMDA coating. This result could be potentially attributed to the different isoelectric point and zeta potential values observed for the two samples in the zeta potential titration measurements (Figure 4). In particular, the higher values observed in the case of the Control + CA/HMDA coating could be indicative of the presence of a higher amount of anionic species that could give rise to a much more favorable interaction with the aqueous medium used for the FRAP assay. On the contrary, the DPPH assay (run in an ethanolic solution) could be favored in the presence of not or less deprotonated species as in the case of the CT + CA/HMDA coating in this condition.

As predictable, the antioxidant activity resulted in negligible changes on the pristine titanium surfaces (Control and CT) in both assays.

The antioxidant potency of both coated substrates persisted after prolonged storage in the air of the materials (up to 12 months), suggesting that the materials could be prepared in advance with respect to their use (Table S1). Interestingly, the antioxidant activity was retained even after a repeated immersion for 1 week in freshly prepared FRAP solutions for up to eight cycles (Figure S4) or for 2 weeks in a DPPH solution for up to four cycles (Figure S5).

### 3.7 Kinetics of the Release of the CA/HMDA Coating from the Titanium Substrates

In order to investigate coating release and chemical stability in aqueous media under simulated physiological conditions, the coated substrates were soaked in PBS (pH = 7.4) at 37 °C up to 28 days in another set of experiments. Aliquots of the medium were withdrawn over time (at 1, 7, 14, and 28 days) and analyzed through the F&C assay. The GAE values determined after the first day of release in PBS (0.74 × 10^–3^ and 0.69 × 10^–3^ μg/mL for Control + CA/HMDA and CT + CA/HMDA, respectively) indicated that the release was faster during the first days and proceeded slowly up to 1 month (Figure S6) if compared to the amounts of the phenolic coating initially deposited on the Control and CT substrates that were equal to 1.2 ± 2.5 × 10^–3^ and 1.6 ± 1.8 × 10^–3^ μg/mL for Control + CA/HMDA and CT + CA/HMDA, respectively, as evaluated by the F&C assay.

In particular, a sustained release (*ca.* 43% with respect to the GAE values estimated before the release experiments) was observed in the case of the CT + CA/HMDA coating after 24 h, whereas this value was higher for Control + CA/HMDA (*ca.* 62% with respect to the GAE values estimated before the release experiments). However, even after the 28 days of release test, detectable amounts of phenolic organic coating, equal to 0.15 × 10^–3^ μg/mL for the Control + CA/HMDA coating and 0.26 × 10^–3^ μg/mL for the CT + CA/HMDA, were revealed by the F&C assay.

### 3.8 Cytocompatibility Evaluation

**Figure 10:**
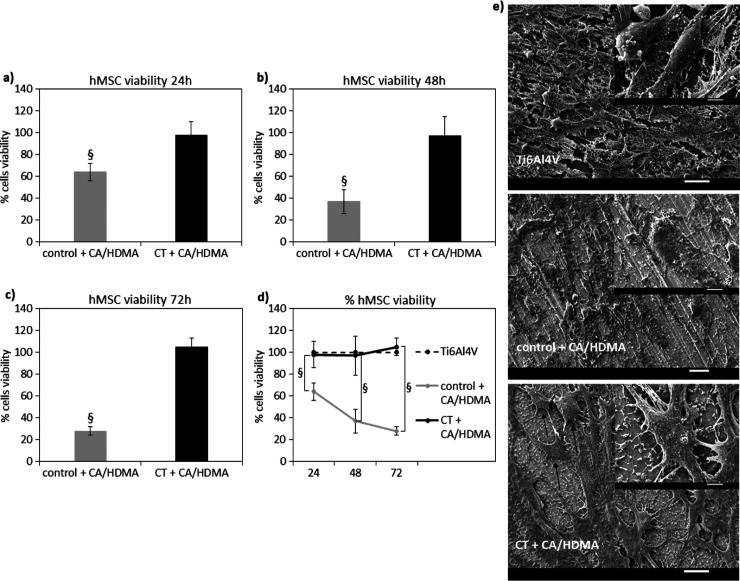
(a–d) Cytocompatibility evaluation by the alamar blue assay after 24 (a), 48 (b), 72 (c) h showed a significant increase of cells’ viability onto CT + CA/HDMA surfaces in comparison to the Control + CA/HDMA ones (*p* < 0.05 indicated by §) as well as an increase of metabolic activity in function of time (d). SEM images of cells cultivated onto specimens’ surface after 72 h (e) demonstrated that cells onto CT + CA/HDMA surfaces displayed a proper morphology comparable to the Ti6Al4V untreated (indicated by arrows) ones, whereas cells onto Control + CA/HDMA showed an apoptotic-like round shape (suggested by the arrows). Bars represent means ± SD, replicates *n* = 3; SEM bar scale = 20 μm (low magnification) and 5 μm (high magnification).

From the perspective of a potential application for bone tissue engineering, the cytocompatibility of the coated materials was assayed toward hMSCs as representative of cells deputed to tissue healing after migrating from niches and unaffected neighboring tissue. Accordingly, cells were cultivated in direct contact with the surfaces of the specimen and observed for 72 h. The results are reported in Figure 10.

The polished Ti6Al4V specimens have been considered the positive control (meaning 100% viability) due to the comparable metabolic activity of the cells seeded onto such surfaces and those cultivated onto the gold standard polystyrene, as reported in Supporting Information Table S2.

In general, the CT + CA/HDMA coating resulted in being highly cytocompatible; in fact, the results of the metabolic activity obtained for the cells cultivated onto coated specimens normalized toward the ones from the Ti6Al4V (considered as 100% viability control) demonstrated that cell viability was >95% at each time-point (Figure 10a–c, *p* > 0.05), showing a sort of plateau between 24 and 48 h and a slight increase at 72 h (Figure 10d). On the contrary, the Control + CA/HDMA coating reported toxicity since the % of viable cells decreased over time in the selected time-points, being always significantly lower with respect to Ti6Al4V and CT + CA/HDMA (Figure 10a–d, *p* < 0.05 indicated by §).

As a confirmation, SEM images (Figure 10e) representative for cells cultivated for 72 h onto specimens’ surfaces established the results of the metabolic assay: in fact, it was possible to appreciate cells at high density well adhered and spread onto the CT + CA/HDMA specimens that were comparable to the images from the Ti6Al4V ones. On the contrary, the round-shaped cells observed on the Control + CA/HDMA specimens are probably mostly in the apoptotic stage where cells take this round shape prior to detaching and dying. The same conclusion was derived from the fluorescent live/dead staining (Figure S7) where the cells cultivated onto the Control + CA/HDMA coating incorporated the red signal from the propidium iodide, thus indicating a compromised membrane integrity representative for the apoptotic stage.

To rationalize the results obtained, wettability cannot be considered as a possible cause of the different cytocompatibilities of the two coatings because they were similar, both the surfaces were hydrophilic, as required for osseointegration, and the contact angles were below the threshold for an anti-adhesive behavior as previously reported by the authors.^60^ Similarly, the differences in terms of topography between the smooth Ti6Al4V and the rough (at the micro-/nanoscale) CT are not sufficient to justify the results; in fact, the presence of micro-/nano-pores due to the surface oxidation was previously demonstrated as being cytocompatible but not sufficient to promote cells’ adhesion nor to boost their metabolic activity.^8^ As debated earlier, it represents mostly a treatment aimed at improving the osteointegration by the formation of a surface titanium oxide layer (300–400 nm thick), presenting a peculiar nanoporous sponge-like morphology favoring ECM deposition and mineralization by adhering osteoblasts.

So, a possible explanation comes from the surface’s chemistry. In fact, from the XPS analysis, it was highlighted that on CT + CA/HDMA specimens, a higher number of amines, mostly aliphatic, had been detected in comparison to the Control + CA/HDMA ones. In particular, the aliphatic amines are essential components of living cells, regulating nucleic acid function, protein synthesis, and the stabilization of membranes; an example was given by Heller et al.,^61^ who demonstrated that the presence of amines favored and improved collagen and fibronectin aggregation into a filament-like structure supporting human osteoblasts and umbilical cord cell adhesion and spread onto Ti substrates. On the contrary, the amines deposited onto Control + CA/HDMA specimens mostly belonged to the heterocyclic group, being commonly related to toxic effects toward living cells. This was demonstrated by the previous literature regarding, for example, human hepatocytes,^62^ where the introduction of the heterocyclic amines in the culture medium determined cells’ apoptosis by oxidative stress, DNA damage, and cytochrome P450 (CYP) activation.

So, according to the results of this screening, the CT + CA/HDMA coating was the most promising treatment in view of a biomedical application of the functionalized titanium substrates.

### 3.9 Scavenging Activity in a Pro-Inflammatory Environment

**Figure 11:**
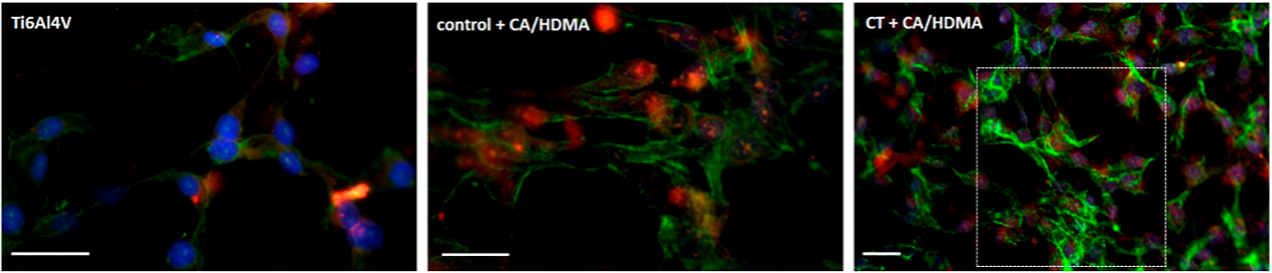
Antioxidant activity. Intracellular CellRox dye stained in red internalized toxic reactive species demonstrating that cells suffer from the oxidative stress due to the generated active species; cells were co-stained with DAPI (blue) and phalloidin (green) to visualize nuclei and the cytoskeleton, respectively, to demonstrate that cells cultivated onto CT + CA/HDMA displayed a higher density and a physiological morphology in comparison to the Control + CA/HDMA and Ti6Al4V ones. Bar scale = 50 μm.

In other experiments, after cells’ seeding, a pro-inflammatory stage was induced by hydrogen peroxide in order to generate high levels of reactive oxygen species in the culture medium as potentially toxic elements for cells. Afterward, the specific CellROX dye internalization was considered to visually investigate if cells suffered from the presence of the active species. Results are reported in Figure 11.

As expected, the absence of scavenging components on the Ti6Al4V specimens prevented from cell protection toward reactive species; as a consequence, few cells were still attached after the H_2_O_2_ treatment (cytoskeletons are stained in green and DAPI in blue) as well as they showed mostly positive results to the CellROX staining (in red). On the contrary, the antioxidant effect of the CA/HMDA coating was well appreciable in the case of the CT + CA/HDMA specimens: first, many cells were still visible on the surface even after the H_2_O_2_ treatment (green and blue signals), and second, some regions (such as the one indicated by the white dashed square) were weakly positive to the red signal of the CellROX, thus suggesting that the coating ensured a scavenging activity useful to prevent internalization of toxic oxygen reactive species into cells. Finally, the results from the Control + CA/HDMA specimens were mostly like the Ti6Al4V ones though the CA/HMDA coating should provide an efficient scavenging activity based on the results of the antioxidant chemical assays; however, the state of suffering of the cells, as expected based on the metabolic assay, could account for this result.

These results are of great interest considering that titanium and its alloys are well-known biocompatible materials largely applied for bone implantation in both orthopedics and dentistry fields,^63^ but they are mostly biologically inert, thus not providing any beneficial hint to the healing process as well as not conferring any protection toward inflammation. Therefore, current research efforts are focused on the improvement of Ti alloys in terms of biological properties.

The authors started from a consideration of the beneficial activities of CA that is known to favor bone repair over bone resorption in the healing process,^64^ providing a pro-regenerative effect comparable to that ensured by biochemical stimulation such as with alkaline phosphatase. In this work, we have implemented a thin film from CA oxidation onto Ti6Al4V surfaces. Indeed, the reactive oxygen species scavenging effect provided by the CA/HMDA coating can be of crucial importance to preserve the healing of the tissue in a critical condition such as within a pro-inflammatory environment.^65^

A pro-inflammatory phase can highly hinder the bone repair process as it promotes a regulation pathway that favors the resorption steps over the healing ones.^66^ In fact, the acute inflammation cascade can originate from several adverse stimuli, but it can worsen into a chronic condition if not properly counteracted by the physiological homeostatic mechanisms. In terms of tissue regeneration, inflammation is known to favor bone resorption and suppress bone formation due to the crosstalk between inflammatory cells (polymorphonuclear leukocytes and cells of the monocyte-macrophage-osteoclast lineage) and those deputed to the healing (mesenchymal stem cell-osteoblast lineage and vascular lineage).^66^ So, introducing anti-inflammatory properties into the implantable Ti6Al4V materials can be very useful to mitigate the inflammation, thus favoring bone repair.

The specific CellROX red dye was largely incorporated by cells cultivated onto Ti6Al4V specimens due to the lack of an active species scavenger; on the contrary, the CA-based coating in the CT + CA/HDMA specimens reduced the amount of reactive oxygen species, thus preserving a higher number of cells (cytoskeleton F-actins stained in green and nuclei in blue) less positive to the red dye internalization.

### 3.10 Antibacterial Efficacy

**Figure 12:**
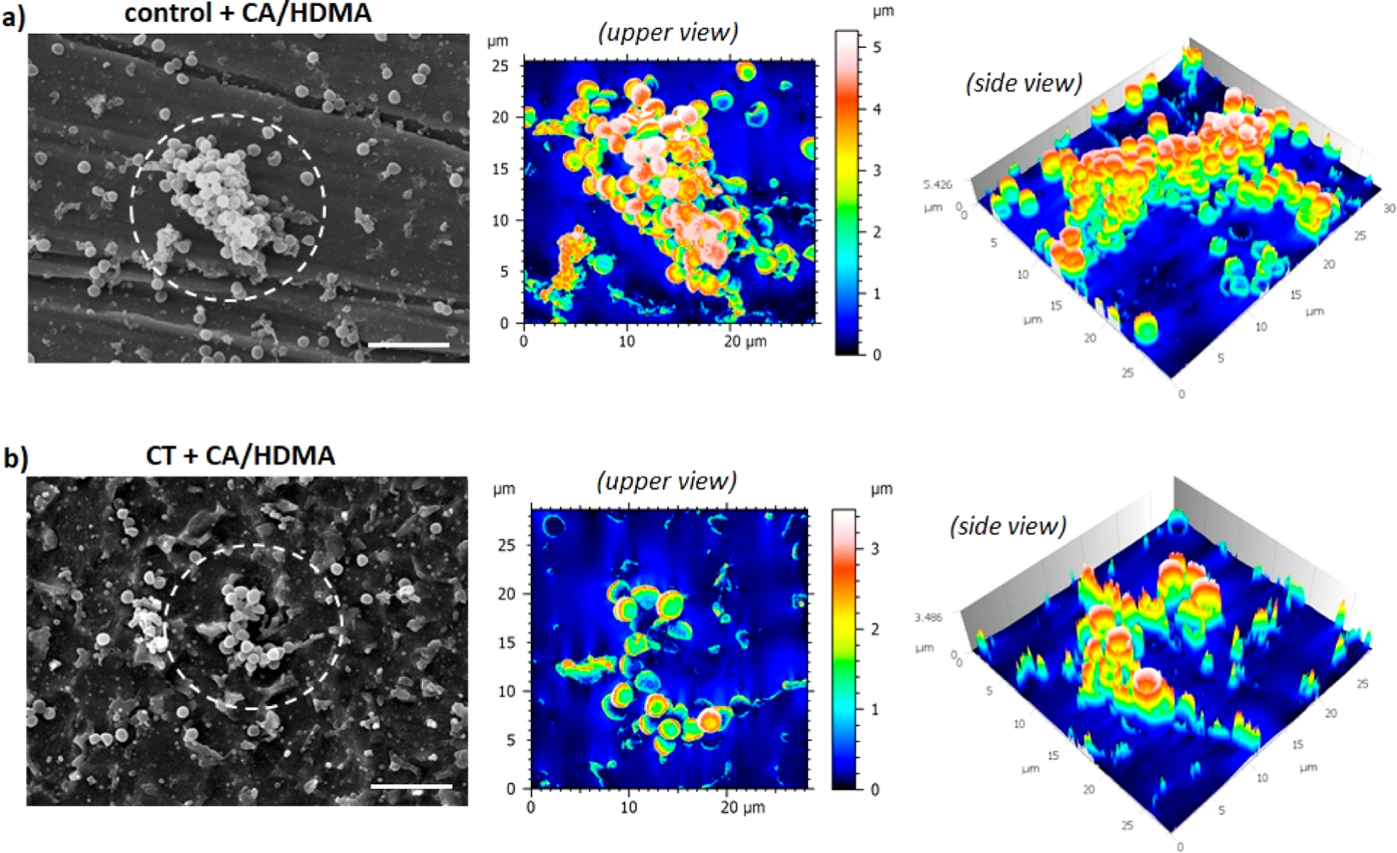
SEM images of the surface-adhered bacterial biofilm onto Control + CA/HDMA (a) and CT + CA/HDMA surfaces. (b) Software-assisted 3D rendering of the thickness (upper and side views—analyzed areas are indicated by the dashed circle). Images revealed that the biofilm grown onto CT + CA/HDMA surfaces was thinner (2–3 μm) than those observed on the Control + CA/HDMA ones (4–5 μm), thus suggesting an antifouling activity in reducing biofilm formation. Bar scale = 5 μm.

As last, the ability of the coated titanium substrates to prevent bacterial infections was investigated. The surface of the bare Ti6Al4V specimens and of the coated ones was directly infected with *Staphylococcus aureus* (*S. aureus*), one of the pathogens mostly related to bone infections in orthopedics where clinical revisions reveal that an *S. aureus* biofilm is frequently strongly correlated with implant failures.^67^ Moreover, the selected strain (ATCC 43300) is a certified MDR strain recommended for drug discovery, therefore an interesting candidate to be tested toward innovative antibacterial strategies due to its resistance to conventional antibiotic therapies. After 24 h of direct infection, the colonization degree was evaluated by means of the metabolic activity of the adhered bacteria and visually checked by the fluorescent live/dead assay; results are summarized in Figure S6. In general, the coatings were unfortunately not effective in preventing the surface colonization from *S. aureus*; in fact, the metabolic activity of the bacteria grown onto the coated surfaces (both Control + CA/HDMA and CT + CA/HDMA) was comparable with the one determined for the uncoated Ti6Al4V (Figure S8a, *p* > 0.05), thus suggesting a similar contamination degree. As a confirmation, the images obtained by the live/dead assay (Figure S8b) showed the presence of viable bacteria (stained in green) colonizing the surface of all the tested specimens. However, an interesting difference is represented by the presence of 3D biofilm-like structures in the Ti6Al4V surfaces. So, to better investigate this anti-fouling activity due to the CA/HDMA coating, SEM was applied to estimate the biofilm thickness by assisted software as reported in Figure 12; by the software 3D rendering, it was possible to rank the biofilm thickness at around 4–5 μm (Figure 12a) for the aggregates observed in the Control + CA/HDMA. The same analysis was then performed on the CT + CA/HDMA specimens (Figure 12b) where biofilm aggregates were found too, but in this case, the displayed maximum thickness measured was at around 2–3 μm, thus suggesting a lower aggregation of bacteria. According to earlier research works,^68,69^ this effect cannot be ascribed to a strong antibacterial activity but to the fact that CA can decrease the biofilm maturation by reducing protein expression^69^ as well as by downregulating the expression of pro-adhesion/aggregation genes such as the agglutinin-like sequences.^68^ In line with this, the effect observed for the CT + CA/HDMA specimens can be due to a combination of the above-mentioned properties of the CA coated on the surface with the micro-/nanotopography obtained after the chemical oxidation that determined the presence of needle-like pillars preventing bacterial adhesion by reducing the anchorage points.^8^

Moreover, some other differences in terms of surface properties can be correlated with the observed impediment of biofilm maturation; the needle-like shape obtained in the CT nanostructure can reduce bacterial proliferation and adhesion by irreversibly harming their membrane and reducing the anchorage-points in the early adhesion phase as previously described by the authors.^8^ It is less likely to correlate biofilm reduction with a decrease of the surface wettability as no significant differences were noticed between the coated materials when the contact angles were measured (Figure 4); it is more probable that a further disturbing element for the biofilm development is represented by the high chemical reactivity of the CT surfaces that is due to the functional −OH groups exposed by the oxide layer formed during the CT. Such high reactivity can bring about an oxidative stress when the functional groups are internalized by bacteria, bringing them to death by metabolic unbalance and DNA damage as it happens, for example, following the administration of polyphenols.^70^

However, there is no doubt that the antibacterial properties of the coatings developed here should be clearly improved in future studies to provide not only a reduction of the biofilm thickness but to prevent bacterial colonization. Moreover, the antibacterial studies will be extended to other Gram-positive and Gram-negative pathogenic strains to confirm a broad-spectrum action.

## 4 Conclusions

Titanium and its alloys are among the most widely employed materials for orthopedic and dental implants due to their good mechanical properties and biocompatibility. In the past years, improvement of the bone integration ability and soft tissue adhesion has been obtained by bioactive coatings, chemical/electrochemical treatments generating bioactive oxide layers, and modification of the surface topography. In this context, the deposition of organic natural coatings on titanium substrates allows for the combination of morphological and chemical/biological stimuli on the same surface because the texture of the titanium substrate will not be masked by the thin coatings.

In this study, the specific film-forming properties imparted by a long-chain diamine (HMDA) to autoxidizing CA were exploited for the design of thin, uniform, and stable films on both polished and CT Ti6Al4V alloys. The coating of CA/HMDA on the titanium surfaces, which was completely characterized from the chemical and physical standpoints by means of the F&C method, fluorescence microscopy, water contact angle measurements, XPS, zeta-potential measurements, and FT-IR, allows us to combine the characteristics of the titanium substrates with the peculiar properties of the organic molecules, obtaining thus bioactive and biocompatible materials. The ability of both coated surfaces to act as antioxidants, thus improving the interfacial properties of titanium implants for osseointegration, is, in fact, of great interest from the perspective of exploitation of these materials in bone tissue engineering.

Particularly encouraging results were obtained for the CT + CA/HDMA substrates, exhibiting high cytocompatibility toward stem cells deputed to promote healing and scavenging activity to protect cells from apoptosis due to toxic active species in a pro-inflammatory environment. However, other strategies should be considered in the future to improve the antibacterial properties of the coating in order to prevent bacterial infections or to deeply investigate the anti-biofilm aggregation effect due to the presence of the CA-based coating as by now only an anti-fouling activity was observed in reducing the thickness of the adhered bacterial biofilm. Moreover, further in vivo studies are required in the future to confirm the coatings’ properties in a more complex environment where other factors not reproducible in vitro can influence the behavior of the devices.

Overall, these results pointed to HMDA as an efficient crosslinking mediator of film deposition from CA. The versatile two-component coating procedure herein reported could be extended to a varied range of naturally occurring phenols as well as different substrates, opening thus new opportunities for the development of novel and biocompatible thin coatings for biomedical applications.

## Supporting Information Available

UV–vis spectra in reflectance modality of the bare and coated Control + CA/HMDA and CT + CA/HMDA samples; ATR–FTIR spectra, DPPH, FRAP assays, and release kinetics of the functionalized titanium surfaces; live/dead fluorescence images of hMSCs, and antibacterial properties’ evaluation toward *S. aureus* on the surface of the controls and coated specimens (PDF)

## Abbreviations

XPS: X-ray photoelectron spectroscopy
FT-IR: Fourier transform infrared
HMDA: hexamethylenediamine
DPPH: 2,2-diphenyl-1-picrylhydrazyl
FRAP: ferric reducing antioxidant power
TPTZ: 2,4,6-tris(2-pyridyl)-*s*-triazine
TPC: total phenolic content
GAE: gallic acid equivalents
FBS: fetal bovine serum
DAPI: 4′,6-diamidino-2-phenylindole
