# Supplementary material for: Deposition of Antioxidant and Cytocompatible Caffeic Acid-Based Thin Films onto Ti6Al4V Alloys through Hexamethylenediamine-Mediated Crosslinking
Source: ACS Appl Mater Interfaces. 2023 Jun 8;15(24):29618–35. doi: 10.1021/acsami.3c05564 (PMC10288441; doi:10.1021/acsami.3c05564)
Supplement: Supplementary file 1 — am3c05564_si_001.pdf [file am3c05564_si_001.pdf]

Supporting Information

Deposition of Antioxidant and Cytocompatible Caffeic Acid-Based Thin Films onto Ti6Al4V Alloys through Hexamethylenediamine-Mediated Crosslinking

Maria L. Alfieri,[†,*] *Giacomo Riccucci,*[‡] *Sara Ferraris,*[‡,*] *Andrea Cochis,*[§] *Alessandro C. Scalia,*[§] *Lia Rimondini,*[§] *Lucia Panzella,*[†] *Silvia Spriano,*[‡] *Alessandra Napolitano*[†]

[†]Department of Chemical Sciences, University of Naples Federico II, Via Cintia 21, I-80126, Naples, Italy.

[‡]Politecnico di Torino, Corso Duca degli Abruzzi 24, 10129, Torino, Italy.

[§]Department of Health Sciences, Center for Translational Research on Autoimmune and Allergic Diseases CAAD, University of Piemonte Orientale, Corso Trieste 15/A, 28100, Novara, NO, Italy

Corresponding author

*Email: marialaura.alfieri@unina.it; sara.ferraris@polito.it

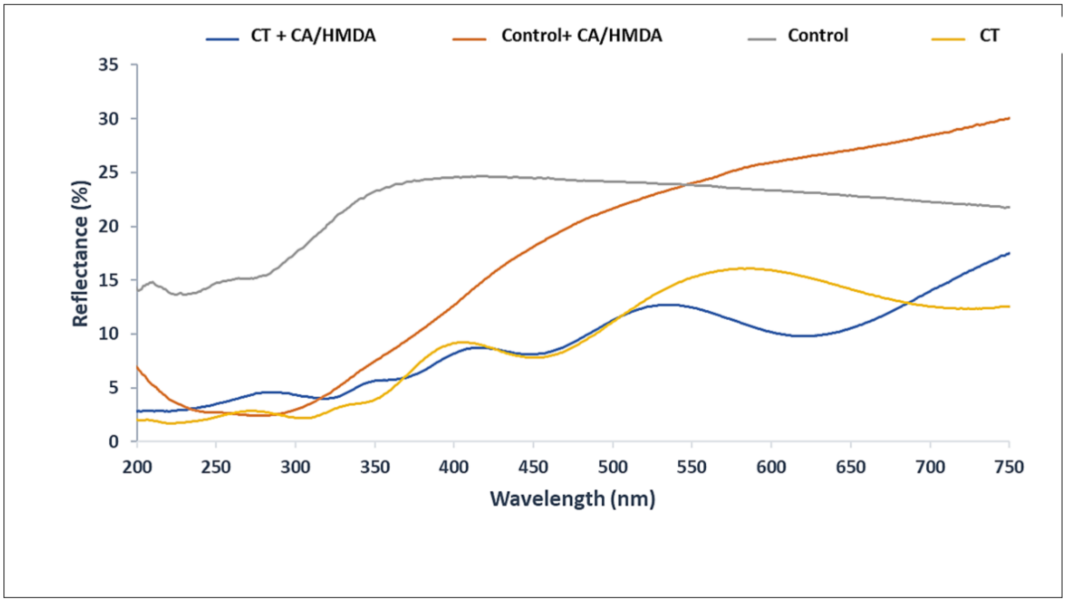

Figure S1. UV-vis spectra in reflectance modality of the bare and coated Control + CA/HMDA and CT+ CA/HMDA samples.

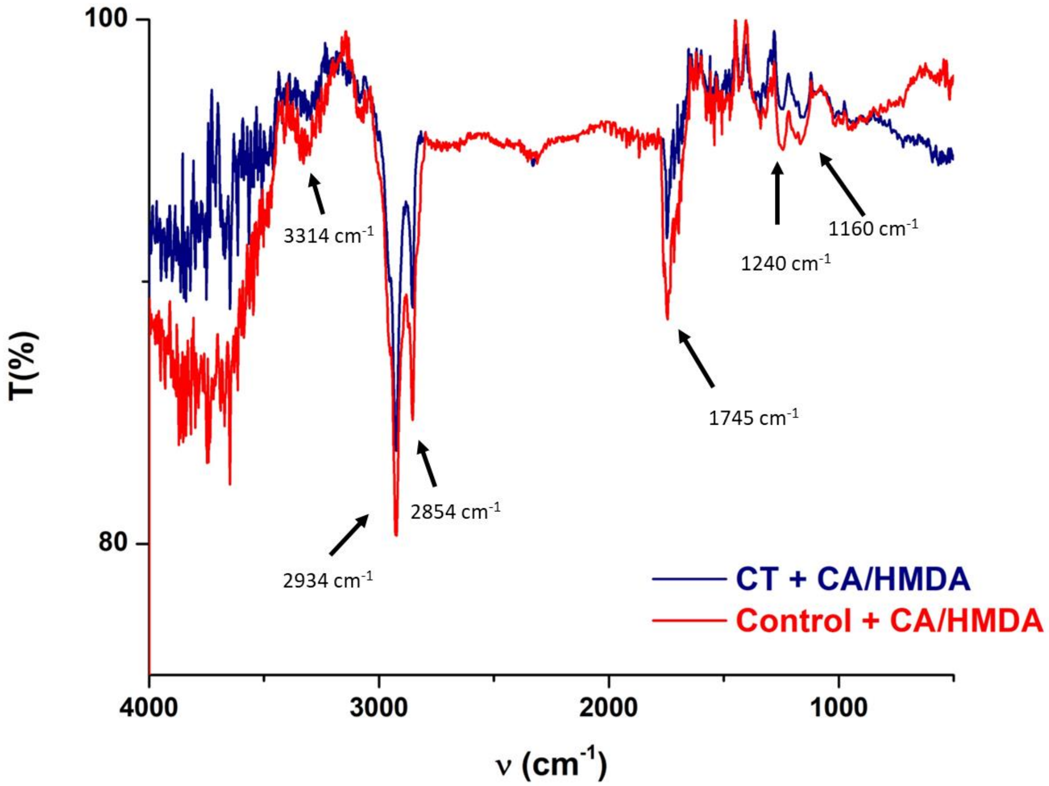

Figure S2. ATR-FTIR spectra of functionalized titanium surfaces: Control + CA/HMDA and CT + CA/HMDA.

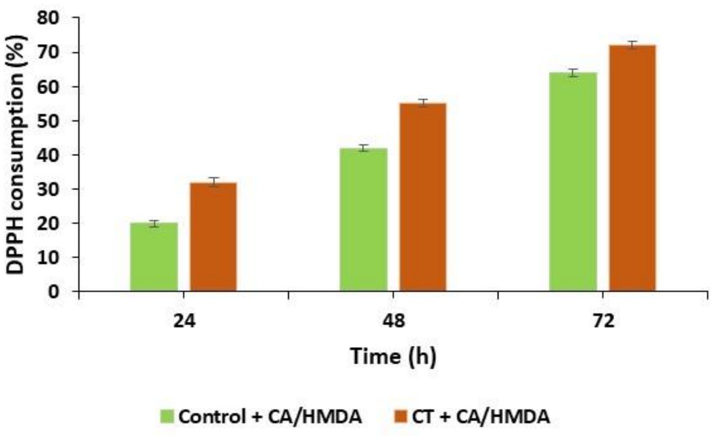

Figure S3. DPPH reduction properties when two coated substrates for type of surfaces (Control+CA/HMDA and CT+CA/HMDA) were dipped in DPPH solution. Reported are the mean ± SD values of two experiments.

Table S1. DPPH and FRAP assays run for 24 h on both Control + CA/HMDA and CT + CA/HMDA coated substrates after they were stored in air for 1 day, 1 week, 1 month or 12 months. Reported are the mean ± SD values of two experiments.

	Control + CA/HMDA		CT + CA/HMDA	
Time of storage	DPPH consumption (%)	Abs Fe^{2+} -TPTZ complex	DPPH consumption (%)	Abs Fe2+ -TPTZ complex
1 day	12 ± 0.51	0.5911 ± 0.020	24 ± 0.80	0.5049 ± 0.030
1 week	11 ± 0.95	0.5895 ± 0.025	24 ± 0.50	0.4973 ± 0.040
1 month	13 ± 1.5	0.5905 ± 0.018	24 ± 0.28	0.5003 ± 0.035
12 months	12 ± 0.44	0.6023 ± 0.007	23 ± 0.78	0.5182 ± 0.015

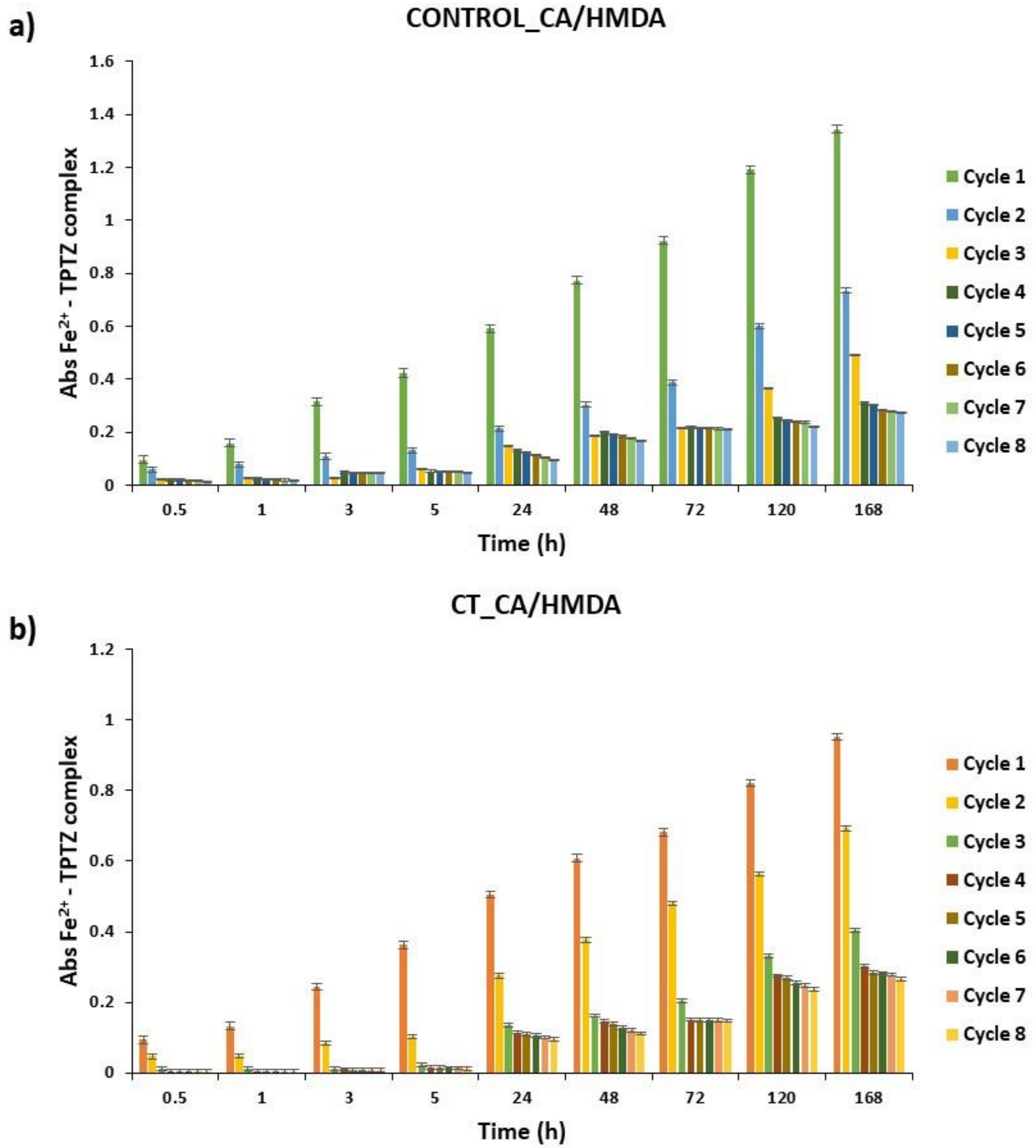

Figure S4. Kinetics of development of the absorption of Fe^{2+}-TPTZ complex over time by immersion of titanium substrates (Control and CT) coated with CA/HMDA films. Shown are the results of triplicates ± SD.

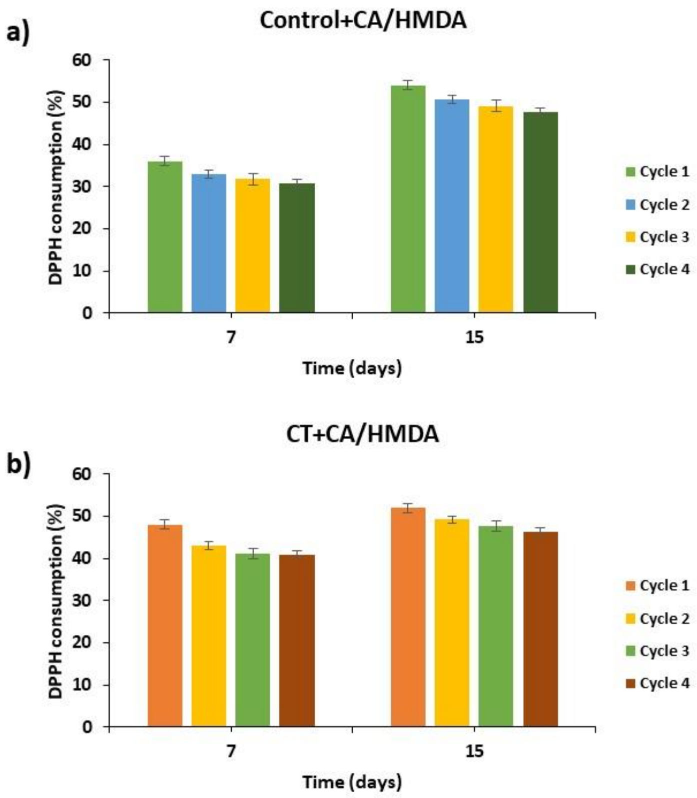

Figure S5. Kinetics of DPPH reduction over time by immersion of titanium substrates (Control and CT) coated with CA/HMDA films. Shown are the results of triplicates ± SD.

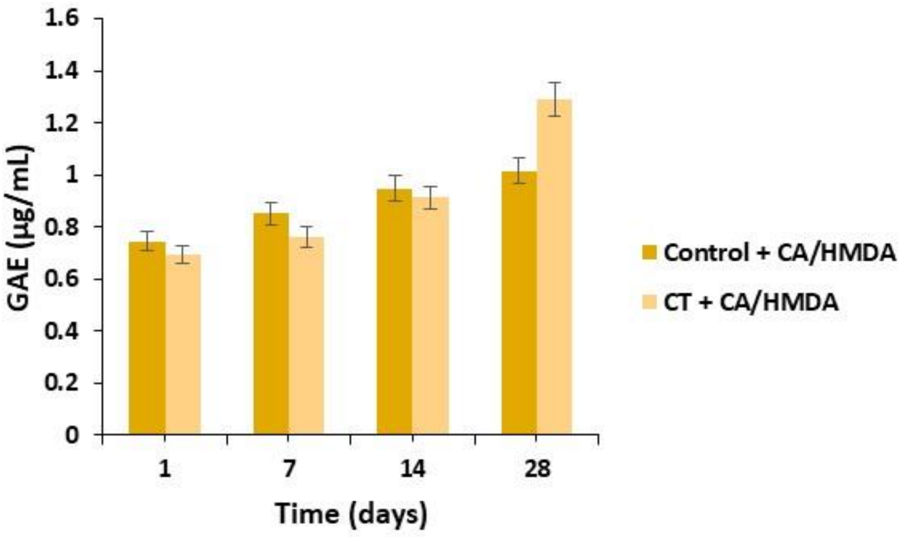

Figure S6. Release kinetics of the CA/HMDA coating from both polished and chemically pre-treated titanium surfaces in phosphate buffer saline (PBS) 1 X (pH 7.4) at 37 °C over 28 days time span. Results were expressed as gallic acid equivalents (GAE) quantified through the Folin and Ciocalteu assay. Reported are the mean ± SD values of three experiments.

Cytocompatibility evaluation

The bulk Ti6Al4V specimens were considered as positive control representing 100% of cells' viability due to the comparable results obtained by the metabolic assay alamar blue in comparison to the gold standard polystyrene as reported in Table S2 below where RFU values and % of viability are presented in function of the selected time-points.

Table S2. Metabolic activity of cells cultivated onto polystyrene gold-standard and Ti6Al4V substrates; results are expressed as relative fluorescent units (RFU) and % of viability normalized towards polystyrene.

specimen	*24h RFU*	*24h %*	*48h RFU*	*48h %*	*72h RFU*	*72h %*
polystyrene	11694 (±766)		14070 (±688)		14091 (±615)	
Ti6Al4V	13053 (±1500)	111% (vs poly)	13095 (±3148)	93% (vs poly)	13476 (±2023)	95% (vs poly)

The images of the Live/Dead assay applied to the cells cultivated for 72h onto the surface of the controls and coated specimens are reported in Figure S7. As expected, the cells grown onto the control Ti6Al4V surfaces are viable (stained in green); regarding the coated ones, the CT + CA/HDMA surfaces resulted as highly compatible as the majority of the cells results as viable, whereas the Control + CA/HDMA surfaces displayed high toxicity as the majority of the cells resulted as not viable (stained in red). Fluorescent images are in line with metabolic and SEM results.

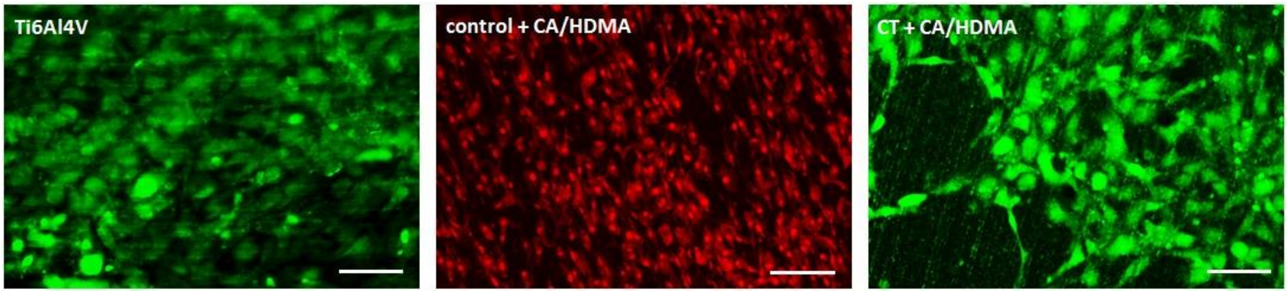

Figure S7. Live/Dead fluorescence images of hMSC cells cultivated for 72h onto specimens' surface (live cells stained in green, dead cells stained in red), bar scale = 125 µm.

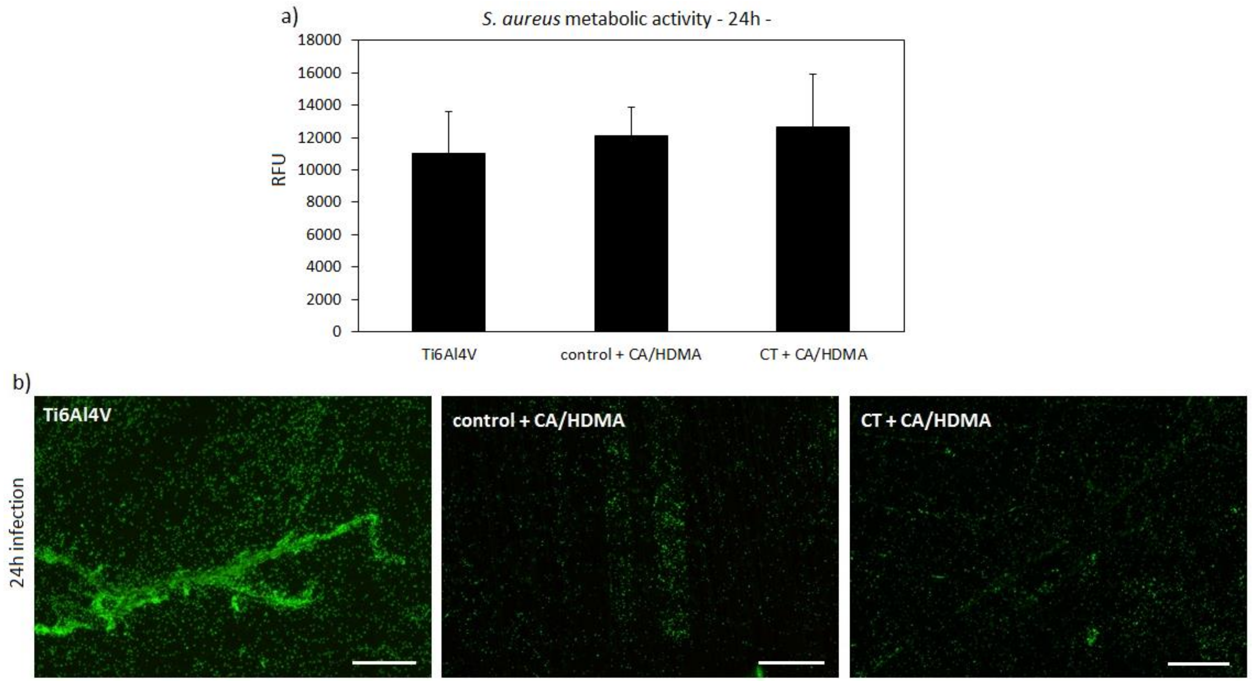

Figure S8. Antibacterial properties evaluation towards *S. aureus*. a) The metabolic activity evaluation of the adhered bacteria revealed no statistically significant differences ($p>0.05$) between control (Ti6Al4V) and coated specimens; b) the visual observation of the viable adhered bacteria (stained in green) confirmed the presence of infection onto all the tested surfaces after 24h. Reported are the mean ± SD values of three experiments. Images bar scale = 100 µm.
